# Subclinical Atherosclerosis Risk Can Be Predicted in Female Patients With Systemic Lupus Erythematosus Using Metabolomic Signatures: An Observational Study

**DOI:** 10.1161/JAHA.124.036507

**Published:** 2025-04-07

**Authors:** Laurel Woodridge, Maria G. Tektonidou, George A. Robinson, Junjie Peng, Leda Coelewij, Lucia Martin‐Gutierrez, Elvira Chocano Navarro, Maura Griffin, Andrew Nicolaides, Coziana Ciurtin, Anisur Rahman, Inés Pineda Torra, Elizabeth C. Jury

**Affiliations:** ^1^ Department of Ageing, Rheumatology and Regenerative Medicine, Division of Medicine University College of London London UK; ^2^ First Department of Propedeutic Internal Medicine “Laiko” Hospital National and Kapodistrian University of Athens Athens Greece; ^3^ Vascular Noninvasive Diagnostic Centre London UK; ^4^ Department of Vascular Surgery Imperial College London UK; ^5^ Department of Vascular Surgery, Nicosia Medical School University of Nicosia Cyprus; ^6^ Andalusian Center for Molecular Biology and Regenerative Medicine (CABIMER) Sevilla Spain

**Keywords:** cardiovascular disease risk, female, metabolomics, SLE, subclinical atherosclerosis, Biomarkers, Lipids and Cholesterol, Translational Studies, Machine Learning, Metabolism

## Abstract

**Background:**

Cardiovascular disease (CVD) is a leading cause of death in women with systemic lupus erythematosus (SLE) due to accelerated atherosclerosis that is not predicted by established CVD risk scores. This study aimed to develop, validate, and test a female‐focused predictive atherosclerosis risk signature based on serum metabolites in patients with SLE.

**Methods and Results:**

Female patients with SLE were assessed for the presence (SLE‐P; n=18) or absence (SLE‐NP; n=26) of subclinical atherosclerosis using vascular ultrasound for carotid/femoral intima‐media thickness. CVD risk was assessed using QRISK3 (which includes SLE diagnosis as a risk factor) and Framingham Risk Score. Serum metabolomics (n≥250) was performed and analyzed using machine learning pipelines. Despite having subclinical atherosclerosis, 44.8% to 100% of patients with SLE‐P had low CVD risk according to QRISK3/Framlingham Risk Score scores. Using a lipid‐focused metabolomic analysis, an improved atherosclerosis risk predictive signature was developed comprising 35 metabolites/5 clinical traits that classified patients with SLE‐P and outperformed CVD risk assessment tools, lipid profiles measured in routine care, and clinical features alone. This “atherosclerosis risk signature” was validated in a second adult female SLE cohort (n=98) that predicted plaque status with moderate accuracy (area under the receiver operating characteristic curve, 0.79). The signature was then refined into a 5‐feature subclinical plaque‐predictive score that not only stratified the combined SLE‐P/SLE‐NP cohorts (n=142; area under the receiver operating characteristic curve, 0.84) but also predicted 3‐year atherosclerosis progression in female postpubertal patients with juvenile‐onset SLE (n=36; area under the receiver operating characteristic curve, 0.79). Finally, the 5‐feature score identified distinct high and low subclinical atherosclerosis risk subgroups in a “real‐world” setting of unscanned adult patients with SLE (n=38).

**Conclusions:**

This atherosclerosis risk score could improve CVD risk assessment/management in female patients with SLE across age. Validation in non‐SLE and healthy cohorts could further substantiate these findings.

**Nonstandard Abbreviations and Acronyms**
ALSPAC: Avon Longitudinal Study of Parents and Children
APPLE: Atherosclerosis Prevention in Pediatric Lupus Erythematosus
BILAG: British Isles Lupus Assessment Group
CIMT: carotid intima‐media thickness
FA: fatty acid
JSLE: juvenile‐onset systemic lupus erythematosus
LR: logistic regression
MESA: Multi‐Ethnic Study of Atherosclerosis
ML: machine learning
SLE‐NP: systemic lupus erythematosus with no subclinical atherosclerosis
SLE‐P: systemic lupus erythematosus with subclinical atherosclerosis
UCLH: University College London Hospitals
UCLH‐40: University College London Hospitals signature
XGB: eXtreme gradient boost

**Clinical Perspective**

**What Is New?**

- A combined metabolite–clinical trait plaque/atherosclerosis risk signature was developed using widely credited machine learning models that correctly classified patients with systemic lupus erythematosus (SLE) with subclinical plaque with high accuracy. This atherosclerosis risk signature was validated in a second external cohort of patients with SLE scanned for subclinical plaque.
- The atherosclerosis signature was refined into a 5‐feature panel that correctly stratified patients with/without subclinical atherosclerosis and identified patients with juvenile‐onset SLE with high versus low atherosclerosis progression.
- In a “real‐world” setting, the 5‐feature atherosclerosis risk panel identified a group of unscanned patients with SLE at potential elevated plaque risk, suggesting that this tool could help improve stratification of at‐risk patients for enhanced cardiovascular disease risk monitoring/treatment.

**What Are the Clinical Implications?**

- Current routine cardiovascular disease risk assessment does not accurately estimate atherosclerosis risk in patients with SLE, meaning that patients who could benefit from more regular monitoring or intervention are not identified.
- Evidence from this study could be used to develop a low‐cost metabolite assay to assess cardiovascular disease risk for use in clinical practice. We highlight several metabolites that can be modulated by diet or lipid‐targeting therapies. Therefore, information presented here could be used to help guide lifestyle and clinical management.

Cardiovascular disease (CVD) is the leading cause of death globally and, despite a greater prevalence in men than women,^1^ ischemic heart disease (16.13%) and stroke (14.26%) are leading causes of death in women (all ages; https://vizhub.healthdata.org/gbd‐compare/).^2^ CVD is particularly prevalent in autoimmune diseases characterized by systemic inflammation and a major cause of death in systemic lupus erythematosus (SLE), a chronic multisystem autoimmune disease with a 90% female prevalence.^3^ The overall risk of CVD has been shown to be increased 50‐fold in women with SLE aged 35 to 44 years,^4^ and CVD accounts for ≈25% of all deaths in SLE, attributed to premature and accelerated atherosclerosis.^5^

The link between SLE and the heightened CVD risk is not completely understood and is complicated by the lack of knowledge about CVD risk in women because a disproportionate amount of CVD research has been conducted in men.^6^ Asymptomatic atherosclerotic plaques appear before the onset of CVD symptoms and have been identified in up to 40% of patients with SLE.^7^ Traditional CVD risk factors, such as dyslipidemia, diabetes, hypertension, and obesity are increased in patients with SLE across age^8^ and are intertwined with SLE‐specific factors contributing to increased CVD risk, including an association with suboptimal disease activity control, antiphospholipid antibodies and SLE treatment type, and subclinical atherosclerosis and CVD.^4^, ^9^

However, CVD risk score assessment tools, such as the Framingham and American Heart Association Atherosclerotic Cardiovascular Disease risk scores, significantly underestimate CVD risk in women and adults with SLE,^10^, ^11^ while estimating this risk in children and young people with juvenile‐onset SLE (JSLE) is even more challenging.^12^ Additional weighting given to a diagnosis of SLE has been adopted in attempts to improve sensitivity, including the QRISK3,^13^ which improves CVD risk detection in patients with SLE.^14^ Notably, not all patients with SLE have dyslipidemia, and patients with SLE often have serum lipid levels within currently defined normal ranges, which could contribute to poor performance of CVD risk assessment scores. Furthermore, vascular screening or other imaging techniques with increased sensitivity for subclinical atherosclerosis detection are not regularly recommended or widely available in SLE care. Thus, there is an unmet need to improve CVD risk assessment in patients with SLE.

Our previous work shows that more detailed analysis of serum metabolite signatures (including complex lipids) can effectively stratify patients with SLE with and without subclinical atherosclerosis.^15^, ^16^ This study now aims to develop, validate, and test a predictive CVD risk signature based on serum metabolites that could identify patients who would benefit from more frequent lipid monitoring or preventative therapies.

## Methods

### Data Availability Statement

Metabolomic data will be made available on publication by request to the corresponding authors.

### Study Participants

**Figure 1:**
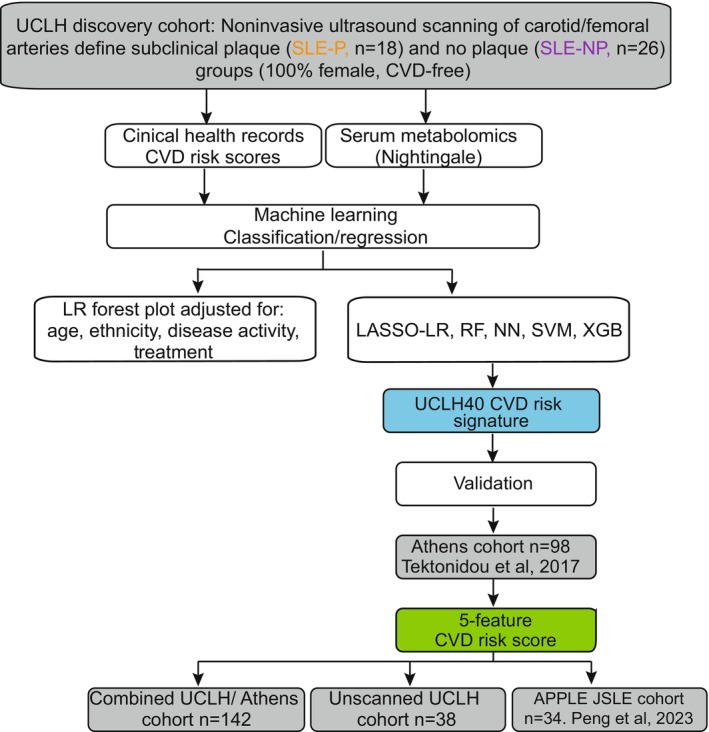
Diagram depicting patient cohorts with SLE and workflow of analysis.^16^, ^21^, ^22^ APPLE indicates Atherosclerosis Prevention in Pediatric Lupus Erythematosus trial; CVD, cardiovascular disease; JSLE, juvenile‐onset systemic lupus erythematosus; LASSO, least absolute shrinkage and selection operator; LR, logistic regression; NN, neural network; RF, random forest; SLE‐NP, systemic lupus erythematosus with no subclinical atherosclerosis; SVM, support vector machine; UCLH, University College London Hospitals; and XGB, eXtreme gradient boost.

See Figure 1 for the study design plan.

#### University College London Hospitals Discovery Cohort

In this cross‐sectional study, consent was obtained from female patients with SLE meeting the American College of Rheumatology classification for SLE^17^ or the Systemic Lupus International Collaborating Clinics criteria^18^ at the time of recruitment (2017/2018) and in accordance with juvenile cohorts, with no previous history of CVD, from an adult rheumatology clinic at University College London Hospitals (UCLH; n=44) and underwent carotid and femoral ultrasound scans.^7^ Patients were stratified by the presence of ≥1 focal plaques in the carotid or femoral arteries (SLE‐P; n=18) and those remaining plaque free (SLE‐NP; n=26). Plaque characteristics assessed by vascular ultrasound are described in Table S1 and in Croca et al.^7^ Clinical and serological measures were recorded at the time of the vascular scan and extracted from UCLH clinical health records (see Table 1 for clinical and demographic information). QRISK3, which includes SLE as a risk factor,^13^ and the Framingham Risk Score^19^ were used to assess CVD risk. Disease activity was assessed using global British Isles Lupus Assessment Group (BILAG 2004) global score with active disease calculated as ≥8.^20^

**Table 1:** Demographics and Clinical Traits Between Patients With SLE‐P and Patients With SLE‐NP in UCLH Discovery Cohort

	SLE‐NP	SLE‐P	*P* value
No.	26	18	
Sex, n (% female)	100	100	
Age, y, mean±SD	44.62±9.11	57.22±6.08	<0.001
Race or ethnicity, n (%)			0.263
Black	5 (19.2)	5 (27.8)	
Asian	2 (7.7)	0 (0.0)	
White	16 (61.5)	13 (72.2)	
Mixed	3 (11.5)	0 (0.0)	
Disease characteristics			
Age at diagnosis, y, mean±SD	28.50±8.70	34.67±10.07	0.04
Disease duration, y, mean±SD	16.12±8.59	22.56±10.60	0.03
Global BILAG 2004, median (IQR)	2.00 (1.00–2.75)	1.00 (0.00–1.00)	0.04*
Anti‐dsDNA, titers, NR <30.0 IU/mL, median (IQR)	46.00 (10.75–165.25)	11.00 (7.00–52.25)	0.13*
Complement 3, NR 0.88–2.01 g/L, mean±SD	0.84±0.31	1.21±0.28	0.01
ESR, (NR 1–20 mm/h, mean±SD	22.29 (13.39)	12.00 (8.94)	0.08
CRP <3.0 mg/L, median (IQR)	1.50 (0.90–3.05)	2.15 (1.05–5.00)	0.23
Lymphocyte, NR 2.2–3.65×10^9/L^, mean±SD	2.37±3.76	1.76±0.80	0.64
Neutrophils, NR 20–7.5×10^9/L^, mean±SD	3.87±1.79	4.19±1.53	0.65
Monocytes, NR 0.2–1.0×10^9/L^, mean±SD	0.46±0.49	0.57±0.20	0.51
Hemoglobin, 115–155 g/L, mean±SD	125.53±7.16	126.78±10.07	0.72
Platelets, NR 150–400×10^9/L^, mean±SD	243.65±49.44	284.22±54.34	0.07
Albumin, NR 34–54 g/L, mean±SD	42.58±2.57	52.00±14.34	0.04
Urea, NR 5–20 mg/dL, mean±SD	4.85±1.17	15.45±22.48	0.10
Creatinine, NR 53–97.2 mg/dL, median (IQR)	62.00 (51.25–66.75)	69.50 (56.25–76.75)	0.19*
Vitamin D, NR >50 nmol/L, mean±SD	62.33±32.05	62.59±25.78	0.98
Albumin, 34–54 g/L, mean±SD	43.23±3.70	42.28±5.56	0.5
Urine protein–creatinine, NR <0.2 mg/mg, median (IQR)	13.00 (9.00–32.25)	16.50 (9.50–34.50)	0.91*
IgG, NR 6.0–16.0 g/L, median (IQR)	13.16 (10.54–14.98)	10.62 (7.86–13.60)	0.08*
IgM, NR 0.4–2.5 g/L, median, (IQR)	0.76 (0.42–1.23)	1.03 (0.46–1.33)	0.71*
IgA, NR 0.8–3.0 g/L, median, (IQR)	2.71 (1.72–3.70)	2.81 (1.95–4.31)	0.48*
CVD‐risk factors at last assessment			
Smoker, ever, n (%)	3 (11.5)	3 (16.7)	0.42
Smoker, current, n (%)	3 (11.5)	2 (11.1)	0.94
Diabetes, n (%)	0 (0)	2 (11.1)	0.09
BMI, NR 18.5–24.9, scan, median (IQR)	25.67 (21.47–28.52)	25.61 (224.48–27.40)	0.54*
MAP, NR 70–100 mm Hg, mean±SD	88.65±9.33	100.69±9.52	<0.001
Total cholesterol, NR <5 mmol/L, mean±SD	4.37±1.04	5.28±0.92	0.004
Triglycerides, NR <3 mmol/L, median (IQR)	0.90 (0.70–1.17)	1.20 (0.83–1.30)	0.11*
HDL‐C, NR >1.2 mmol/L, mean±SD	1.64±0.50	1.79±0.56	0.35
LDL‐C, NR <3 mmol/L, mean±SD	2.29±0.89	2.98±0.88	0.02
Cholesterol‐HDL ratio, NR <6, median (IQR)	2.70 (2.32–3.25)	3.20 (2.35–3.58)	0.21*
Treatment (at time of scan)			
Hydroxychloroquine use, n (%)	17 (65.4)	9 (50)	0.32
Statin use, n (%)	9 (34.6)	2 (11.1)	0.09
ACE inhibitor use, n (%)	7 (26.9)	7 (38.9)	0.41
Aspirin use, n (%)	3 (11.54)	3 (16.7)	0.64
Immunosuppressant use, n (%)	12 (46.2)	6 (33.3)	0.41
Prednisolone dose, mg, median (IQR)	5.00 (0.00–5.94)	5.00 (0.00–5.00)	0.86*
B‐cell depletion, n (%)	7 (26.9)	7 (38.9)	0.41*

All clinical traits were tested for normality; means±SDs were reported for normally distributed data, and median and IQR reported for nonnormally distributed data. Statistical significance assessed by *t* tests. ACE indicates angiotensin‐converting enzyme; BILAG, British Isles Lupus Assessment Group; BMI, body mass index; CRP, C‐reactive protein; CVD, cardiovascular disease; ESR, erythrocyte sedimentation rate; HDL‐C, high‐density lipoprotein cholesterol; IQR, interquartile range; LDL‐C, low‐density lipoprotein cholesterol; MAP, mean arterial pressure; NR, normal range; SLE‐NP, systemic lupus erythematosus with no subclinical atherosclerosis; SLE‐P, systemic lupus erythematosus with subclinical atherosclerosis; and UCLH, University College London Hospitals.

* Wilcoxon rank‐sum for nonparametric testing.

#### Athens Validation Cohort

Female patients with SLE (N=98) fulfilling ≥4 of the American College of Rheumatology classification criteria for SLE^17^ and without clinically diagnosed CVD were recruited in 2012 from the Rheumatology Unit, First Department of Propaedeutic Internal Medicine at Laiko Hospital, National and Kapodistrian University of Athens, Greece. Patients underwent vascular ultrasound scans for subclinical atherosclerotic plaques (described in Tektonidou et al^21^) (SLE‐NP, n=77; SLE‐P n=21). Further information is found in Supplementary Methods.

#### JSLE Cohort

Female patients with JSLE enrolled in the APPLE (Atherosclerosis Prevention in Pediatric Lupus Erythematosus) trial between 2006 and 2009 (ClinicalTrials.gov Identifier NCT00065806; Chief investigator, Laura E. Schanberg).^22^ Female patients with JSLE (n=34: postpuberty; age: 16.44±2.44 [range, 11–20]; 100% female patients) in the placebo group were stratified according to carotid intima‐media thickness (CIMT) progression over 3 years (high progression, n=19; low, n=15) described in Peng et al.^16^

#### Test Unscanned Cohort (UCLH)

An additional cross‐sectional cohort of women with SLE (N=38) with no prior CVD history attending UCLH Rheumatology Clinic were recruited between 2015 and 2019. Clinical features were recorded at the time of serum sampling.

#### Study Approvals

All patients provided informed written consent/assent according to the Declaration of Helsinki. All patient information was anonymized or pseudo‐anonymized in accordance with relevant data protection legislation (EU General Data Protection Regulation [https://www.eugdpr.org/] and UK Data Protection Bill, 2018). This study was approved by the London–City and East Research Ethics Committee of the National Health Service 15‐LO‐2065, London–Harrow Research Ethics Committee (REC11/LO/0330) (UCLH cohorts); the Institutional Review Board of Laiko Hospital, Athens, Greece (Athens cohort), and local institutional review board approval from the 21 Childhood Arthritis and Rheumatology Research Alliance sites in North America (APPLE cohort).

### Metabolomics

Sera from patient cohorts were analyzed using the Nightingale Health nuclear magnetic resonance metabolite platform comprising ≥250 metabolites, including absolute concentrations, percentages, and ratios of lipoprotein composition: apolipoproteins, very‐low‐density, intermediate‐density (IDL), and high‐density (HDL) lipoprotein particles of different sizes ranging from chylomicrons and extremely large, very large, large, medium, small, and very small; the composition of each lipoprotein class, including concentrations of total lipids, phospholipids, cholesterol, cholesterol esters, free cholesterol, and triglycerides; other metabolites include amino acids, glycolysis‐related metabolites, and ketone bodies (Nightingale Health, https://research.nightingalehealth.com/). Table S2 provides a metabolite list.

### Machine Learning–Derived Metabolomics Signature Curation

Classification and regression methods were applied to metabolomic data: univariate logistic regression (LR), least absolute shrinkage and selection operator LR, random forest, neural network, support vector machine and eXtreme gradient boost (XGB) (described in Supplementary Methods). Briefly, models were optimized to specific parameters^23^ and 10‐fold cross‐validation was conducted. Model performance was assessed using accuracy (area under the receiver operator characteristic curve [AUROC]), sensitivity, specificity, and an F1 score (derived from recall and precision). Classification models that performed with an accuracy AUROC >0.7 were included in developing a CVD risk signature. A UCLH signature (UCLH‐40) was derived from the top variables appearing in ≥3 top‐performing (AUROC >0.7) models discriminating SLE‐P from SLE‐NP, which was validated in the Athens‐Validation cohort using the same pipeline.

### SLE Atherosclerosis Risk Score

A weighted score for predicting subclinical atherosclerosis‐risk was derived from the validated UCLH‐40 using Youden statistic and Autoscore analysis using the most significant features (see Supplementary Methods). Hierarchical clustering of metabolite concentrations was used to stratify an independent cohort of adult patients with SLE (UCLH‐unscanned, N=38, 100% women).

## Results

### SLE Patients With Subclinical Atherosclerotic Plaque Are Classified as Low CVD Risk Using Established CVD Risk Scores

CVD‐free women with SLE (N=44) (UCLH discovery cohort) were analyzed for the presence of atherosclerotic plaques in their carotid and femoral arteries using vascular ultrasound (Supplementary Methods). Patients that had a plaque (SLE‐P, N=18/44 [41%]) were compared with patients that remained plaque free (SLE‐NP, N=26/44 [59%]) (Figure 1 for study plan; Table 1 for demographic information). Patients with SLE‐P were significantly older and had a longer disease duration compared with patients with SLE‐NP but had a lower disease activity at the time of scan, assessed using the BILAG 2004 index, and had higher complement 3 and albumin levels. No differences were seen with other inflammatory disease markers or treatment between the groups (Table 1).

CVD risk was also assessed between the patient groups. No difference was found in prevalence of diabetes, smoking status, or body mass index. However, patients with SLE‐P had elevated mean arterial pressure (MAP) and higher serum total cholesterol and low‐density lipoprotein (LDL) levels, although for most patients they were within currently established normal ranges (Table 1). Of note, only 2 patients with SLE‐P were treated with statins. Furthermore, many patients with SLE‐P were not flagged as high risk when assessed using widely used and validated CVD risk scores, the Framingham Risk Score, and QRISK3 (Table 2). Using the Framingham Risk Score, no patients with SLE‐P were classified as high risk. The QRISK3 score, which includes SLE diagnosis as a risk factor, identified 44.4% (8/18) of patients with SLE‐P as low risk, 16.7% (3/18) as intermediate risk, and only 33.3% (6/18) as high risk, despite having subclinical atherosclerosis assessed by vascular ultrasound scanning (Table 2). These results confirm that currently used CVD risk calculators underperform at correctly identifying individuals with subclinical atherosclerosis in SLE.^11^, ^24^

**Table 2:** CVD Risk Scores Do Not Predict Subclinical Plaque in Many Patients With SLE

CVD risk scores		All patients with SLE (N=44) n (%)	SLE‐P (N=18) n (%)	SLE‐NP (N=26) n (%)
Framingham Risk Score				
Low risk, %	<10	44 (100)	18 (100)	26 (100)
Moderate risk, %	10–20	0	0	0
High risk, %	>20	0	0	0
Missing		0	0	0
QRISK3 score				
Low risk, %	<10	28 (63.6)	8 (44.4)	20 (76.9)
Moderate risk, %	10–20	7 (15.9)	3 (16.7)	4 (15.4)
High risk, %	>20	8 (18.2)	6 (33.3)	2 (7.7)
Missing		1 (2.3)	1 (5.6)	0

Statistical significance of cardiovascular risk scores between SLE‐P and SLE‐NP. Cardiovascular risk scores using Framingham Risk Score 10‐year calculator with lipid laboratory results (lipid labs) and without (simple) and QRISK3. A score could not be calculated if data were missing at the time of recording or unknown. Number of patient scores indicated by n; % reflects percentage of patients with score out of total patients with score obtainable. CVD indicates cardiovascular disease; SLE, systemic lupus erythematosus; SLE‐NP, systemic lupus erythematosus with no subclinical atherosclerosis; and SLE‐P, systemic lupus erythematosus with subclinical atherosclerosis.

### Metabolomic Assessment Defined a Novel Signature Associated With Subclinical Plaque in Female Patients With SLE

Serum metabolomic analysis was conducted using a high‐throughput nuclear magnetic resonance platform (≥250 metabolites, Table S2) to define metabolomic signatures associated with subclinical atherosclerosis in the UCLH discovery cohort. Univariate LR identified 69 statistically significant metabolites distinct between patients with SLE‐P and patients with SLE‐NP (*P*<0.05) after controlling for ethnicity, age, age at diagnosis, disease duration, body mass index, BILAG, MAP, smoking status, diabetes, and current treatments (hydroxychloroquine, statins, angiotensin‐converting enzyme inhibitors, immunosuppressants, and prednisolone use and dosage [mg]; Figure S1). Notably, several molecules linked to atherosclerosis were significantly different between SLE groups, including apolipoprotein B, total cholesterol, total cholesteryl esters, free cholesterol, and remnant cholesterol, although no difference was seen for triglycerides (Figure S1). Several nonstandard lipoprotein subclasses were also significantly differentially expressed in patients with SLE‐P. These included HDL, LDL, IDL, and very‐low‐density lipoprotein molecules of varied sizes and with different lipid content, as well as sphingomyelins and fatty acids (FAs), including total FA, linoleic acid, omega‐6, polyunsaturated FA, and saturated FA. The nonessential amino acid glycine was significantly lower in patients with SLE‐P compared with patients with SLE‐NP (Figure S1).

**Figure 2:**
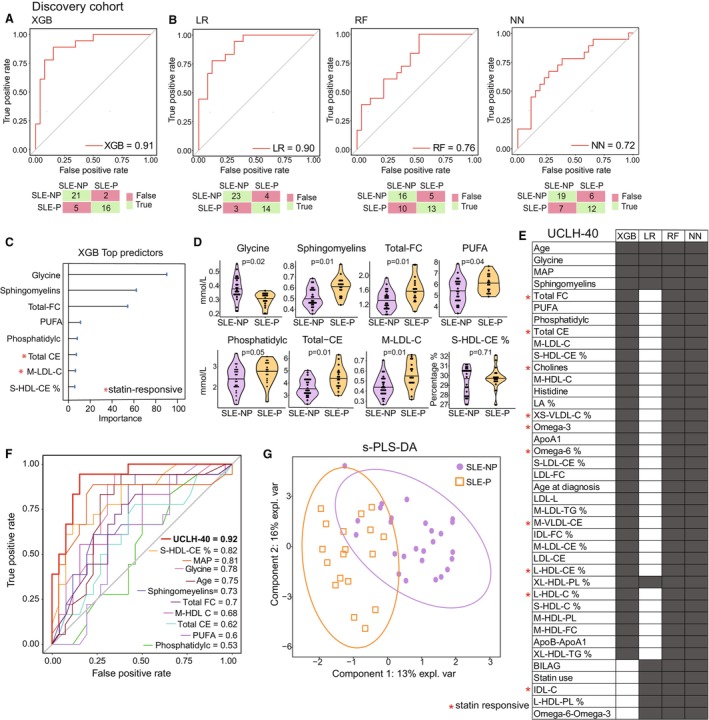
Subclinical plaque can be predicted using a combined serum metabolite/clinical trait signature identified using machine learning models. **A**, AUROC and confusion matrix for predicted group membership (true=green, false=red) for XGB model in SLE‐P vs SLE‐NP. **B**, AUROC and confusion matrices for LR, RF, and NN models. **C**, Metabolite feature importance for XGB model ranked by importance. *=responsive to statin‐treatment.^25^ **D**, Violin plots for top features in XGB model (from **A**) with statistical significance from LR forest plot indicated (Figure S1). **E**, UCLH‐40 signature curated from features predicting plaque status with high importance across all high performing (ROC>0.7) models (**A** and **B**), ranked by importance in XGB model. Black rectangle indicates prevalence of feature in model. *=responsive to statin‐treatment.^25^ **F**, AUROC curves for UCLH‐40 signature and top individual features from XGB model (**A**) predicting SLE‐P vs SLE‐NP classification. **G**, sparse partial least squares discriminant analysis model using UCLH‐40 signature to classify SLE‐P from SLE‐NP. ApoA1 indicates apolipoprotein A1; ApoB, apolipoprotein B; AUROC, area under the receiver operating characteristic curve; CE, cholesterol ester; FC, free cholesterol; IDL‐C, intermediate‐density lipoprotein cholesterol; LDL‐FC, low‐density lipoprotein free cholesterol; L‐HDL‐PL, large high‐density lipoprotein phospholipid; LR, logistic regression; MAP, mean arterial pressure; M‐HDL‐C, medium high‐density lipoprotein; M‐HDL‐FC, medium high‐density lipoprotein free cholesterol; M‐LDL‐C, medium low‐density lipoprotein; M‐LDL‐TG, medium low‐density triglyceride; NN, neural network; PUFA, polyunsaturated fatty acid; RF, random forest; ROC, receiver operating characteristic; S‐LDL‐CE, small low‐density lipoprotein cholesterol ester; S‐HDL‐CE, small high‐density lipoprotein cholesterol ester; SLE‐NP, systemic lupus erythematosus with no subclinical atherosclerosis; SLE‐P, systemic lupus erythematosus with subclinical atherosclerosis; S‐VLDL‐C, small very‐low‐density lipoprotein cholesterol; UCLH‐40, University College London Hospitals signature; XGB, eXtreme gradient boost; and XS‐VLDL‐C, extremely small very‐low‐density lipoprotein cholesterol.

To further evaluate features associated with plaque status, 5 machine learning (ML) models were applied to the metabolomic data combined with clinical features. Due to the biological relatedness of several molecules, homology reduction (≥95%) was applied to the metabolite panel to prevent model overfitting. In total, 121 metabolites and 14 clinical traits were incorporated in the classification and regression models. The performance of 4 of 5 models achieved high accuracy (AUROC >0.7; Table S3). The XGB model showed the highest discrimination accuracy (AUROC, 0.91; F1 score, 0.73), correctly identifying 89% (16/18) of SLE‐P cases (Figure 2A). Three other models (LR, random forest, neural network) performed with similarly high accuracy (AUROC, 0.9, 0.76, 0.72, respectively; Figure 2B). Top‐performing XGB model features were ranked by importance (Figure 2C) and showed statistically significant differences between patients with SLE‐P and patients with SLE‐NP (*P*<0.05), including reduced glycine and elevated sphingomyelin, total free cholesterol, total cholesterol esters, medium‐density LDL cholesterol (M‐LDL‐C), polyunsaturated FAs, and phosphatidylcholine concentrations in SLE‐P (Figure 2D).

Ranked feature importance was compared across all top‐performing models (XGB, LR, random forest, neural network) revealing 35 metabolites and 5 clinical traits associated with subclinical plaque status in SLE in ≥3 high performing models (Figure 2E). Several of these metabolites were statin responsive in previous pharmacological studies,^25^ including free cholesterol (total), total cholesterol esters (esterified cholesterol), IDL‐C, and choline (Figure 2E). These features and clinical traits were combined to generate an SLE‐plaque‐associated signature named UCLH‐40, which could predict plaque status with high accuracy (AUROC, 0.92; Figure 2F). Importantly, the predictive power of the UCLH‐40 was greater than the top individual features, including glycine (AUCOC, 0.78), sphingomyelin (AUROC, 0.73) and S‐HDL‐cholesterol esters % (AUROC, 0.82), hypertension (MAP: AUROC, 0.81) and age (AUROC, 0.75; Figure 2F), as well as clinical serum lipids (total cholesterol, LDL, HDL, triglycerides, cholesterol:HDL ratio) (AUROC, 0.69) that are used in standard clinical practice to assess CVD risk (Figure S2A). However, a model comprising only clinical features that would be available to most clinicians in a real‐world outpatient clinical setting (age, smoking, blood pressure, diabetes, and disease activity) was also able to stratify patients with SLE‐P from patients with SLE‐NP with high accuracy (AUROC, 0.879; Figure S2B), although this model did not perform as well as the metabolite model (Figure 2A) and does not provide the opportunity to monitor CVD risk interventions since most features are nonmodifiable. Finally, the UCLH‐40 was able to distinguish patients with SLE‐P from patients with SLE‐NP using sparse partial least squares discriminant analysis, confirming the efficacy of this novel signature (Figure 2G).

### The UCLH‐40 Was Validated in a Second Cohort of Adult Patients With SLE Scanned for Subclinical Plaque

**Figure 3:**
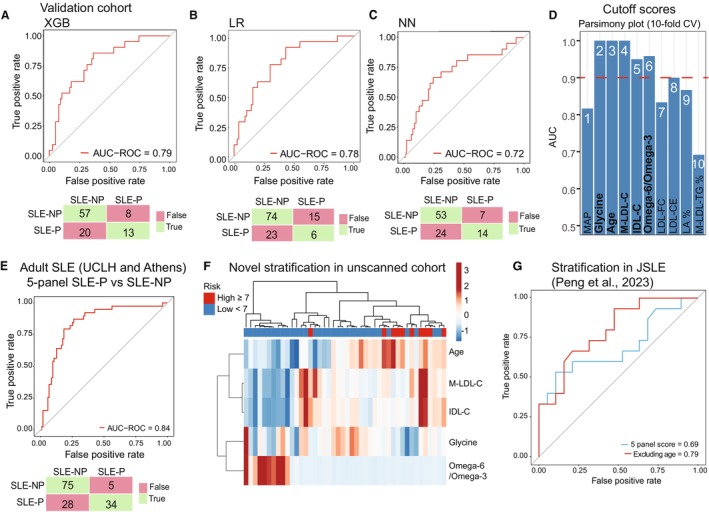
Validation of UCLH‐40 signature and development of CVD risk prediction tool for SLE. **A** through **C**, AUROC and confusion matrix (true predictions=green, false predictions=red) of UCLH‐40 signature in Athens validation cohort (n=98) for SLE‐P (n=21) vs SLE‐NP (n=77) using (**A**) XGB model, (**B**) LR model, and (**C**) NN model. **D**, Parsimony plot showing top features in UCLH‐40 signature that predict SLE‐P using RF analysis ranked by importance. Red dotted line indicates 0.9 AUROC cutoff. **E**, Using 5‐panel score (≥7, high risk) to predict plaque status in combined UCLH Discovery and Athens Validation cohorts. AUROC and confusion matrix. **F**, Heat map showing unsupervised hierarchical clustering in unscanned UCLH cohort grouped by 5‐panel score: High CVD risk, ≥7/low CVD risk, <7. Arms represent correlations between features (row) and samples (column) using correlation clustering. Tightest cluster plotted first. **G**, AUROC indicating accuracy of 5‐panel score in female JSLE cohort^16^ to predict high vs low CIMT progression using XGB models with and without age. AUROC indicates area under the receiver operating characteristic curve; CIMT, carotid intima‐media thickness; CVD, cardiovascular disease; JSLE, juvenile‐onset systemic lupus erythematosus; LR, logistic regression; NN, neural network; RF, random forest; SLE‐NP, systemic lupus erythematosus with no subclinical atherosclerosis; SLE‐P, systemic lupus erythematosus with subclinical atherosclerosis; UCLH‐40, University College London Hospitals signature; and XGB, eXtreme gradient boost.

The subclinical plaque‐associated signature, UCLH‐40 (Figure 2F), was validated in a separate cohort of female CVD‐free patients with SLE (Athens validation cohort: SLE‐P, n=21; SLE‐NP, n=77; Table S4).^21^ Subgroups were matched clinically, except the SLE‐P group was older and had a longer disease duration, consistent with the UCLH cohort (Table 1). Using the top‐performing XGB model, patients with SLE‐P were classified from patients with SLE‐NP with a moderate accuracy (AUROC, 0.79) correctly identifying 71.4% (70/98) of patients (Figure 3A, Table S5). Two other models also performed with good discrimination accuracy (LR, AUROC, 0.78; neural network, AUROC, 0.72; Figure 3B and 3C, Table S5), validating the use of the UCLH‐40 in a globally independent group of patients.

To improve the signature for potential future clinical application, features in the UCLH‐40 were ranked by importance (Figure S2C), and cutoff thresholds were calculated using the Youden statistic (Supplementary Methods). Metabolites with an AUROC value ≥0.90 were selected, creating a simplified 5‐feature panel that was more clinically translatable comprising age, glycine, M‐LDL‐C, IDL‐C, and omega‐6/omega‐3 ratio (Figure 3D and Table S6). A points‐based system was derived to account for the weighted contribution of each feature in the panel (n=4 metabolites, 1 clinical trait) across a points‐based scale (Table S7). To assess the accuracy of this score to identify patients with plaques (assumed to have a higher CVD risk metabolic profile), the 5‐panel score was applied to the combined adult scanned cohorts (UCLH Discovery and Athens Validation). Plaque status was predicted with 84% (34/39) accuracy (Figure 3E), and 90% (n=93/103) of patients with SLE‐NP were stratified into the low‐CVD‐risk group (Table S8). The 5‐panel score was also applied to historic serum metabolomic data obtained from the UCLH discovery patient cohort collected 5 years before this study using an older version of the metabolomic platform (reported in Coelewij et al^15^) and was able to stratify patients into high‐ and low‐CVD‐risk groups corresponding to plaque status: 71% (15/21) of patients in the high‐CVD‐risk group had subclinical plaque by vascular ultrasound,^15^ and 88% (44/50) in the low‐CVD‐risk group were plaque free (Figure S2D), thus confirming the stability of the signature over time.

To mimic a real‐world clinical experience, a third independent cohort of patients with SLE, who had not undergone vascular scans for the presence of subclinical plaques (UCLH test unscanned cohort), was assessed using the 5‐panel score. Applying the optimal atherosclerosis risk threshold score (≥7) in this unscanned cohort identified distinct groups of patients at potential high and low CVD risk (Figure 3F). Interestingly, there were few clinical and demographic differences between patients in the 2 groups, except the high‐risk group was older (as expected from our analyses) and had significantly elevated LDL‐C cholesterol, a clinical CVD risk marker (Table 3). These results suggest that the 5‐panel score could identify potential high‐risk groups which, once confirmed in a clinical study, support its possible application in a clinical setting, where patients identified as high atherosclerosis risk could benefit from a follow‐up ultrasound scan or targeted management for elevated CVD risk.

**Table 3:** High Versus Low Risk in Unscanned UCLH Cohort

	Low	High	*P* value
n	28	10	
Metabolite risk score (high risk ≥7), mean±SD	4.21±1.45	7.90±0.74	<0.001
Age, mean±SD	43.89±14.00	54.30±11.56	0.04
Disease duration, y, mean±SD	18.60±12.32	19.40±10.41	0.86
Race or ethnicity, n (%)			0.44
Asian	7 (25)	4 (40)	
White	9 (32.14)	3 (30)	
Black	9 (32.14)	2 (20)	
Mixed	3 (10.71)	1 (10)	
Disease activity at the time of sampling			
BILAG 2004, mean±SD	4.46±5.82	3.30±4.74	0.57
SLEDAI, mean±SD	3.32±4.17	2.80±1.99	0.71
Active (SLEDAI ≥6 or BILAG ≥8), n (%)	9 (31.14)	3 (30)	0.9
LLDAS, n (%)	14 (50)	4 (40)	0.6
Anti‐dsDNA, titers, NR <30.0 IU/mL, median (IQR)	52.00 (10.25–97.25)	124.50 (7.50–174.50)	0.73
Complement 3, NR 0.88–2.01 g/L, mean±SD	1.03±0.24	1.11±0.25	0.4
Lymphocyte count, mean±SD	1.14±0.67	1.36±0.59	0.37
ESR, NR 1–20 mm/h, median (IQR)	16.00 (7.50–30.50)	8.00 (7.50–16.00)	0.4
CRP, NR <10 mg/L, median (IQR)	1.65 (0.88– 2.10)	2.40 (1.00–5.20)	0.48
Treatment			
Rituximab, n (%)	7 (25)	3 (30)	0.76*
Hydroxychloroquine, n (%)	16 (57.14)	5 (50)	0.71
Hydroxychloroquine dose, mg, median (IQR)	0.00 (0.00–50.00)	0.00 (0.00–0.00)	0.89*
Prednisolone, n (%)	20 (71.43)	7 (70)	0.93
Prednisolone dose, mg, mean±SD	6.10±3.15	5.67±4.04	0.84
MMF, n (%)	4 (14.29)	3 (30)	0.28
Azathioprine, n (%)	5 (17.86)	2 (20)	0.88*
Methotrexate, n (%)	2 (7.14)	0 (0)	0.39*
CVD risk factors			
Smoking, ever, n (%)	1 (3.57)	0 (0)	0.56
Smoking, current, n (%)	1 (3.57)	1 (10)	0.45
Diabetes, n (%)	0 (0)	0 (0)	
Total cholesterol, mean±SD	4.03±1.85	5.33±1.96	0.07
Clinical LDL‐C, mean±SD	2.13±1.13	3.09±1.46	0.04
HDL‐C, mean±SD	1.30±0.75	1.62±0.51	0.22
Total triglyceride, mean±SD	1.03±0.72	1.02±0.42	0.98

Clinical features in unscanned UCLH cohort split by metabolite risk groups (high vs low). All clinical traits were tested for normality; means±SDs were reported for normally distributed data, and median and IQR reported for nonnormally distributed data. Statistical significance assessed by *t* tests. Hypertension (abnormal, yes or no), systolic/diastolic. BILAG indicates British Isles Lupus Assessment Group; BMI, body mass index; CRP, C‐reactive protein; CVD, cardiovascular disease; ESR, erythrocyte sedimentation rate; HDL‐C, high‐density lipoprotein cholesterol; IQR, interquartile range; LDL‐C, low‐density lipoprotein cholesterol; LLDAS, low lupus disease activity state; MMF, mycophenolate mofetil; NLR, neutrophil lymphocyte ratio; SLEDAI, Systemic Lupus Erythematosus Disease Activity Index; and UCLH, University College London Hospitals.

* Wilcoxon rank‐sum for nonparametric testing.

### Atherosclerosis Risk Signatures Developed in Adult SLE Can Also Predict Atherosclerosis Progression in Young Patients With JSLE

The 5‐panel score associated with subclinical plaque was also assessed in a cohort of younger patients with JSLE, who typically have greater disease severity and treatment burden, and an even greater relative CVD risk compared with adults.^26^ The 5‐panel score was applied to serum metabolomic profiles assessed in a subgroup of female patients with JSLE from the placebo arm of the APPLE trial (assessing the efficacy of atorvastatin on 3‐year atherosclerosis progression in JSLE based on CIMT assessment; n=34; 100% female patients; postpuberty; average age, 16 years).^16^ The top‐performing model in adult patients with SLE (XGB) performed less well in identifying patients with JSLE with high versus low CIMT progression over 3 years (AUROC, 0.69). However, as age is a significant driver of CVD risk in adult SLE cohorts and the JSLE cohort were much younger, this could explain why the score was less sensitive in predicting CIMT progression. Removing age improved the performance accuracy (AUROC, 0.79; Figure 3G), suggesting that concentrations of glycine, omega‐3/6 ratio, M‐LDL‐C, and IDL‐C were strong metabolite features associated with CVD risk (plaque status or CIMT progression) in both adult and juvenile SLE.

Finally, to explore whether metabolites in the 5‐panel score could potentially be associated with CVD outcomes, metabolomic and incidence data from the UK Biobank were examined in the general population (non‐SLE).^27^ Concentrations of the 4 metabolites, omega‐6/omega‐3, glycine, IDL‐C, and M‐LDL‐C, had various effect sizes associated with the incidence of myocardial infarction, stroke, and peripheral vascular disease (all populations considered; Figure S3A through S3C), suggesting potential utility of this approach in non‐SLE/healthy populations.

## Discussion

Patients with SLE have an elevated CVD risk through accelerated atherosclerosis, and CVD is one of the leading causes of death in SLE,^1^ yet assessment of CVD risk and evidence indicating an appropriate treatment for CVD in SLE is lacking. The need for improved CVD risk monitoring is supported by the observation that, across a 5‐year follow‐up, 5 of 36 (13.8%) patients with baseline plaque developed coronary disease in the wider UCLH SLE cohort.^28^ This study focused on trying to identify female patients with SLE who are at elevated CVD risk associated with subclinical atherosclerosis, which is not recognized by existing established CVD risk scores.^10^, ^11^, ^13^ We developed an integrated serum metabolite signature that accurately predicted subclinical atherosclerosis status in patients with SLE using serum metabolomics and clinical traits that was validated across several patient cohorts with SLE across age to determine atherosclerosis risk in SLE. The signature better stratified patients with subclinical atherosclerosis compared with existing CVD risk assessment tools and individual biomarkers (such as routinely measured serum lipids). A clinically translatable 5‐panel atherosclerosis risk signature comprising low glycine concentration and omega‐3/omega‐6 ratio, elevated M‐LDL‐C and IDL‐C, and older age was used to determine novel SLE subgroups associated with elevated CVD risk. This signature could be used either with or without age to identify patients with JSLE with an elevated CIMT progression and thus elevated CVD risk. Therefore, this study showed that CVD risk assessment could be improved using nonstandard serum lipids and other metabolites combined with readily available clinical information. However, this study was limited by the small sample size of the discovery cohort, and while the subsequent validation cohort was larger and supported the original findings with moderate accuracy, these findings would benefit from further validation in other cohorts to ensure their validity and minimize risk of model overfitting, including non‐SLE cohorts.

In addition, although the identified atherosclerosis risk signature was able to predict the presence of subclinical atherosclerosis with moderate accuracy across different cohorts, our study was not designed to provide information about the likelihood and type of CVD event a patient may have in the future. Rather, we wanted to find potential new biomarkers to detect increased CVD risk, which warrants improved clinical monitoring (such as referral for vascular ultrasound scanning) and management (lifestyle modification/lipid‐lowering therapy) in a population that is currently not monitored closely for this risk despite its high prevalence. Our approach, to assess both carotid and femoral arteries for atherosclerotic plaques, is supported by the observation that plaques can be detected in the absence of intima‐media thickening in patients with SLE^29^; therefore, a comprehensive assessment of different vascular sites is advantageous. Furthermore, higher intima‐media thickness or presence of plaque can predict future coronary artery disease or stroke in SLE.^30^ Peripheral artery disease is less studied in SLE, although patients are at increased risk,^31^ and future studies need to address the efficacy of serum metabolites for these patients.

The pathogenesis underlying SLE means that SLE itself is an independent risk factor for premature atherosclerosis in the absence of traditional risk factors.^32^ Therefore, it is unsurprising that existing CVD risk assessments that rely on measures developed in the general population using traditional CVD risk factors and scores have been criticized for underestimating risk in SLE.^11^ The limited application of existing scores may be explained by a male bias in CVD research, whereas SLE has a female predominance (female:male ratio, 9:1). Traditional risk factors, age, and hypertension^1^ were associated with subclinical atherosclerosis in SLE, whereas diabetes, smoking status, and obesity status were less important. Clinical serum cholesterol level measurements classified many patients with plaque as having a low‐CVD‐risk profile, thus illustrating the poor performance of these measures in the context of SLE. Various attempts to improve the sensitivity of CVD risk assessment have included SLE adaptation using disease‐specific weighting,^14^ but the European League Against Rheumatism recommendations for CVD risk management in rheumatic diseases, including SLE and antiphospholipid syndrome, have not endorsed any specific tool due to limited evidence.^33^ Interestingly, we also identified that a model including only clinical features normally available to most clinicians in the clinic, including age, smoking, MAP, diabetes, and disease activity, could also identify patients with subclinical atherosclerosis. Since both the composite clinical score and the combined metabolomic/clinical score have similar improved performance compared with currently available tools,^10^, ^11^ both signatures could be explored interchangeably in clinical practice for patient stratification. The added benefit of the combined clinical and metabolomic score is that it provides more information about modifiable and druggable targets, including more complex lipids, going beyond current serum lipid assessments. Comparatively, the clinical score alone has few modifiable factors where aging and diabetes are nonmodifiable, and lupus disease will be treated in the same way for all patients. Thus, while the clinical score predicts CVD risk in SLE, it has less value as a routine measure of CVD risk management, as it is limited to identifying changes in only hypertension and disease activity, which are already regularly monitored in routine practice.

Recent advances in the resolution of metabolomics and nuclear magnetic resonance technology have increased understanding of how lipoprotein molecular weight and composition contribute to atherosclerosis. Lipid networks are complex and interdependent, which requires use of advanced statistical approaches that can compensate for biological relatedness, including homology reduction and ML. ML has proven effective in several clinical settings, including atherosclerosis detection and progression,^34^ as well as CVD event prediction.^35^ Importantly, in this study (MESA [Multi‐Ethnic Study of Atherosclerosis] cohort; 52.6% female participants),^35^ classification of patients with subclinical plaque from those without was improved using a multicohort validated signature developed using several widely adopted ML models, suggesting a panel of markers would be more accurate for clinical use compared with individual biomarkers alone. This study established that the 2013 American Heart Association/American College of Cardiology CVD Pooled Cohort Equations risk calculator, used in the general population to assess 10‐year risk of atherosclerotic cardiovascular disease (defined as heart attack, CHD death, or stroke), achieved an area under the curve of 0.68 for the female group, increasing to an area under the curve of 0.76 during validation. Using an ML model improved the accuracy of the predictions (based on standard risk factors) with an area under the curve of 0.92 for the female subgroup. Of note, in this study only 1 type of ML model was used (support vector machine) rather than the several models used in our study.

Better patient stratification using more sensitive lipid/metabolite analysis established here could help improve the efficacy of clinical trials targeting CVD in SLE whereby incorrect assessment of atherosclerosis severity at baseline could affect the overall outcome of the trial.^16^ Statin trials in SLE have shown mixed outcomes whereby atorvastatin use failed to improve coronary artery calcium deposition, CIMT, or carotid plaque^36^ or CIMT progression in children with SLE.^22^ However, any improvement in some patients may have been diluted by lack of effect in others who would not benefit from statin treatment due to having a low CVD risk before treatment initiation. Enrichment trials in which initial recruitment is informed by choosing patients at higher CVD risk may be more informative in the future, but currently, the rationale for statin use in SLE remains uncertain. Not all patients with SLE have dyslipidemia, and patients with SLE often have serum lipid levels within currently defined normal ranges; therefore, statins are not routinely prescribed.^37^ Furthermore, there is a reluctance to use statins in women of childbearing age due to concerns about pregnancy. Few patients in the UCLH cohort were treated with lipid‐lowering statins, yet several metabolites identified in this analysis are responsive to statin treatment^25^ and were shown to be important predictors of plaque status, supporting that some patients could benefit from statin therapy. Retrospective analysis of statin trial outcomes using the 5‐panel score stratification (developed in adult and juvenile cohorts) at baseline could provide additional validation and a more sensitive evaluation of statin use for CVD progression across all age groups, an approach used to assess baseline lipid profiles and atherosclerosis progression in JSLE previously.^25^

The CVD profile in patients with JSLE compared with patients with SLE is distinct and often associated with worse outcomes,^12^ so the observed differences between juvenile and adult cohorts in prediction accuracy using the 5‐panel score are somewhat expected. In addition, the JSLE cohort was stratified by CIMT progression over the course of the trial, rather than the presence of atherosclerotic plaques as in the adult cohorts. Follow‐up studies could include vascular ultrasound scanning of patients with JSLE to confirm the compatibility of these groupings. Of note, other atherosclerotic plaque measures were obtained during vascular ultrasound scan of the adult UCLH cohort, including total plaque area and number of plaques. These measures provide further information on plaque burden and atherosclerosis severity and have been shown to be strongly associated with greater CVD risk in the wider UCLH cohort^7^, ^28^ and therefore could be used to interrogate markers of atherosclerosis progression in JSLE.

Key features associated with plaque status in SLE in several analyses and in a previous study^15^ included glycine deficiency, which could lead to diminished capacity for triglyceride‐rich very‐low‐density lipoprotein uptake by macrophages resulting in elevated triglyceride content.^38^ Glycine is used in the synthesis of several biologically important compounds, including purines and glucose.^39^ It exerts anti‐inflammatory and antioxidative effects^40^, ^41^ and has been inversely associated with traditional cardiovascular risk factors, including obesity, hypertension, and diabetes.^42^, ^43^, ^44^ Plasma glycine was inversely associated with risk of acute myocardial infarction and higher plasma glycine was associated with a more favorable baseline lipid profile and lower prevalence of obesity, hypertension, and diabetes.^45^ Interestingly, large‐scale epidemiological analyses found that glycine is genetically associated with lower CHD risk, which may be partly driven by changes in blood pressure.^46^ Metabolism of glycine and its precursor amino acid, serine, are central to many aspects of cell metabolism, including lipid metabolism and the de novo production of sphingolipids.^47^, ^48^ Interestingly, glycine and serine are reduced in patients with metabolic syndrome, and aberrant serine homeostasis causes glycine and serine deficiency. Furthermore, dietary serine supplementation mitigates dyslipidemia in diabetic mice, supporting a link with metabolism of sphingolipids.^49^ Sphingomyelins were also identified as a top discriminating feature in UCLH‐40 and were increased in SLE‐P. However, other sphingolipids, such as ceramide, were not present in the metabolomic panel. A more detailed analysis of the glycine, serine, and sphingolipid metabolism pathways, including analysis of ceramides involved in regulating apoptosis and sphingomyelin synthesis^50^ and other processes implicated in CVD, could further elucidate a role of sphingolipid dysregulation in CVD risk in SLE.

High‐plaque classification accuracy was also associated with lower omega‐3/omega‐6 ratio, whereby the imbalance between polyunsaturated FAs (including omega‐3) and saturated fats (eg, omega‐6) leads to dysregulated metabolic homeostasis, hyperlipidemia, and inflammation.^51^ In the wider population, this metabolite was considered a strong predictor of acute myocardial infarction. These components are modulated by diet, which differs geographically. Although these markers were validated in global cohorts (United Kingdom, Athens, and United States), additional investigation in non‐Western populations would provide additional confirmation for their diagnostic value. IDL‐C and M‐LDL‐C were important predictors of plaque stratification in this study, which are established proatherogenic drivers of atherosclerosis due to their lower density compared with atheroprotective HDL molecules.

This study had some limitations. First, it was initiated as a clinical study between 2011 and 2013 when the scans were first performed (reported in Croca et al^7^) with no formal power calculation implemented. The data presented here are based on an analysis of patients who consented to a second scan and who also agreed to providing a blood sample. Therefore, this analysis is based on a pragmatic approach, in a real‐world outpatient clinic setting and resulted in a relatively small sample size and imbalance between SLE subgroups, which is, unfortunately, common when investigating patients with rare conditions.^52^ Although this study used multiple independent cohorts to identify and validate a female‐focused CVD risk signature, patients with plaque typically represented around 30% to 40% of patients,^7^ which meant that SLE subgroups were naturally imbalanced. In this case, although the number of independent samples is relatively low, the high quality and the high dimensionality of the data (250 metabolites, demographic and clinical information) does favor an ML approach.^53^ An additional benefit of using both ML and statistical analysis in this data set is that ML models mostly use a nonparametric approach by not making assumptions about the distribution of the data and can capture nonlinear patterns/relationships more effectively compared with linear statistical models. To overcome small sample size in this study, we used several widely used ML models including random forest, neural network, support vector machine, XGB, and LR with least absolute shrinkage and selection operator penalization, and to reduce potential bias and overfitting (associated with small sample size) we used 10‐fold cross‐validation.^54^ Finally, by combining the data from multiple ML models, we identified the metabolites/features that were most important for stratifying patients with SLE‐P from patients with SLE‐NP in >1 model. Importantly, we show that we were able to validate and refine the CVD risk signature in multiple different cohorts, which adds confidence to our findings. In addition, this study focused on female patients with SLE. A cohort of male patients with SLE was not available for this study, and it is possible that male patients with increased CVD‐risk may not be identified using this female‐focused signature. Unfortunately, due to the nature of the international cohorts analyzed in this study, different validated clinical assessment tools were used to assess disease activity of the patients (BILAG 2004 and SLE Disease Activity Index 2000), although these scores have been shown to be comparable in clinical practice to assess disease activity.^55^ When the clinical data for SLE‐P and SLE‐NP were compared in both the discovery (assessed by BILAG 2004) and validation (assessed by SLE Disease Activity Index 2000) cohorts, most patients had low disease activity, and disease activity was not highlighted as a discriminatory feature in the ML models between patients with SLE‐P versus patients with SLE‐NP.

Future studies testing the 5‐panel atherosclerosis‐risk score developed here to assess CVD risk in patients without SLE and healthy controls should be considered. Several large cohorts including the UK Biobank, which has CIMT data on a subset of patients,^56^ and historical cohorts such as the CheckPoint and ALSPAC (Avon Longitudinal Study of Parents and Children; United Kingdom) studies^57^ have metabolomic data available using the same nuclear magnetic resonance platform (Nightingale Health). Interestingly, data from the UK BioBank Nightingale Health Atlas^27^ did provide some evidence that the atherosclerosis risk–associated metabolites in the 5‐panel score could also be associated with incidence of cardiovascular and peripheral vascular events in the general population, although more granular examination of a female‐only and age‐matched cohort is needed for more accurate conclusions to be drawn.

In conclusion, this study has illustrated the need for more effective patient stratification to identify female patients with SLE with heightened atherosclerosis risk who could benefit from targeted strategies to address this risk, including aspirin, statins, or other lipid‐lowering therapies earlier in their life. Lipid dysregulation characterized SLE and subclinical atherosclerosis in this study, reflecting a key subclinical stage of atherosclerosis development that could be targeted as a preventative strategy in at‐risk patients. In the absence of routine vascular ultrasound or CIMT monitoring, these results suggest that this atherosclerosis risk signature could be applied in clinical practice to assess CVD risk in patients with SLE across age. Identified patients could be prioritized for monitoring and preventative treatment where standard clinical assessment has failed.

## Sources of Funding

BBSRC London Interdisciplinary Biosciences PhD consortium (BB/M009513/1), National Institute for Health Research University College London Hospital Biomedical Research Centre (BRC531/III/IPT/101350) and the Rosetrees Trust and grant PID2021‐126077OB‐I00 from MCIN/AEI/10.13039/501100011033 and FEDER, UE, 10.13039/501100008530 – European Regional Development Fund. The funders had no role in the design and conduct of the study; collection, management, analysis, and interpretation of the data; preparation, review, or approval of the manuscript; and decision to submit the manuscript for publication.

## Disclosures

The authors declare no competing interests.

## Supporting information

Data S1
Tables S1–S7
Figures S1–S2
