# Supplementary material for: Subclinical Atherosclerosis Risk Can Be Predicted in Female Patients With Systemic Lupus Erythematosus Using Metabolomic Signatures: An Observational Study
Source: J Am Heart Assoc. 2025 Apr 7;14(8):e036507. doi: 10.1161/JAHA.124.036507 (PMC12132899; doi:10.1161/JAHA.124.036507)
Supplement: Supplementary file 1 — Data S1 Tables S1–S7 Figures S1–S2 [file JAH3-14-e036507-s001.pdf]

SUPPLEMENTARY MATERIAL

Subclinical atherosclerosis risk can be predicted in female patients with systemic lupus erythematosus using metabolomic signatures.

Laurel Woodridge PhD[1], Maria G Tektonidou PhD[2], George Robinson PhD[1], Junjie Peng MSc[1], Leda Coelewij PhD[1], Lucia Martin-Gutierrez MSc[1], Elvira Chocano Navarro MSc[1], Maura Griffin PhD[3,] Andrew Nicolaides DSc[3,4,5], Coziana Ciurtin PhD[1], Anisur Rahman PhD[1], Inés Pineda Torra PhD[1,6]#, Elizabeth C Jury PhD[1]#

# Equal contribution and joint senior authors

[1] Division of Medicine, University College of London, 5 University Street, London, WC1E 6JF, UK.
[2] First Department of Propedeutic Internal Medicine "Laiko" Hospital**,** National and Kapodistrian University of Athens, 17 Agiou Thoma Str., 115 27, Goudi, Athens, Greece.
[3]Vascular Noninvasive Diagnostic Centre, London, U.K.
[4]Department of Vascular Surgery, Imperial College, London, U.K.
[5]Department of Vascular Surgery, Nicosia Medical School, University of Nicosia, Cyprus.
[6] Andalusian Center for Molecular Biology and Regenerative Medicine (CABIMER) Avda. Americo Vespucio 24. Edif. CABIMER 41092, Sevilla, Spain.

Supplementary Methods

Table S1. Atherosclerotic plaque characteristics- UCLH Discovery Cohort
Table S2. Metabolite Abbreviations
Table S3. Performance metrics for ML serum metabolomics analysis
Table S4. Athens validation cohort – SLE patient clinical and demographic characteristics
Table S5. Validation of UCLH metabolite signature in Athens cohort
Table S6. Youden scores for metabolite panel
Table S7. Metabolite panel points system.
Table S8. 5-panel risk scores in adult SLE:

Figure S1: Forest plot showing metabolomics univariate LR analysis of UCLH Discovery Cohort.
Figure S2. Validation of atherosclerosis-risk scores.
Figure S3: Assessment of metabolites in atherosclerosis risk score using UK Biobank data

Supplementary Methods

Vascular ultrasound scans (Discovery cohort)

Information regarding scanning is described in detail in [1]. In brief, vascular ultrasound scans of the common carotid artery, carotid bulb, carotid bifurcation, common femoral artery, and femoral bifurcation were performed bilaterally using the Philips IU22 ultrasound computer and the L9-3 MHz probe. Intima media thickness (IMT) measurements were performed using QLAB Advanced Quantification Software® version 7.1 (Philips Ultrasound, Bothell, USA) twice within the course of 5 years. Plaque was defined as "a focal thickening >1.2 mm that encroaches into the arterial lumen as measured from the media-adventitia interface to the lumen interface" [2]. Patients who had at least one region fulfilling this description were included in the group with plaque (SLE-P). Patients were split into groups: with plaque reported in at least one carotid or femoral artery by the second scan (SLE-P, n=24) and those remaining plaque-free at both scans (SLE-NP, n=27). Information about carotid and femoral vascular scans performed on SLE patients in the Validation (Athens) cohort are described in[3]. Information about vascular scans performed in the JSLE (APPLE trial) patient cohort are described in [4,5].

Power Calculation

This study was initiated as a clinical study between 2011 and 2013 when the scans were first performed (reported in [1]) with no formal power calculation implemented. The data presented here is based on an analysis of patients who consented to a second scan (2017/2018) and who also agreed to provide a blood sample, n=44. Therefore, this analysis is based on a pragmatic approach, in a real-life outpatient clinic setting.

An *a priori* analysis was performed based on the top metabolites identified in historic serum metabolomic data obtained from the UCLH discovery patient cohort collected five years prior to this study using an older version of the metabolomic platform (Brainshake platform - now Nightingale platform, reported in Coeleweij ATVB 2021 [6]). To detect an effect size of at least 0.9 with p<0.05 at 80% power the sample size is 7-18 samples/group (for 85% power, n=8-20 samples/ group). Calculations done with Power: using the two lipids with significant changes (p<0.05) (L-VLDL-FC and XXL-VLDL-PL were used for these calculations. Therefore, our expected cohort size is sufficiently powered to detect differences between the different SLE subgroups.

Clinical data

UCLH Discovery cohort and UCLH Test (unscanned*):* Clinical values were taken at the time of sample extraction or time of the scan where necessary using clinical health records. For UCLH-scanned, values were obtained for routine measures, including anti-double stranded DNA antibody (ds-DNA) levels, complement component (C)3, whole blood count, erythrocyte sedimentation rate (ESR), SLE disease activity determined by the British Isles Lupus Assessment Group (BILAG) Index-2004 [7]. Coronary artery calcification was not measured.

Routinely measured serum lipid measures (triglycerides, LDL, HDL, total cholesterol) were obtained within 1 year of scan as they are not routinely screened in SLE. Mean arterial pressure (MAP) was estimated via the following formula using diastolic (DP) and systolic pressure: (SP) DP+1/3(SP-DP) as described in[8] who showed a relationship between MAP and CHD in 39000 Chinese subjects. Body mass index (BMI) was calculated as (Weight (kg))/(Height(m^2))

Athens Validation cohort: Disease duration was not available, so this trait was excluded and systemic lupus erythematosus disease activity index (SLEDAI) score (active disease =>6) [9] was used instead of BILAG to assess disease activity. These measures have been shown to be comparable [10].

Test unscanned cohort (ULCH): Standard clinical serum cholesterol measures were not obtained during clinic visit. Instead, Nightingale measures were used to represent clinical lipid measures as they use the same molecular unit.

CVD Risk scores

Values were calculated using the respective R packages. For QRISK3 [https://github.com/YanLiUK/QRISK3.git][11,12], missing values were indicated as 0 or if too much data was missing, available data was entered manually [https://qrisk.org/] which applied default settings to any unknown traits. For CVrisk [https://github.com/vcastro/CVrisk] for Framingham Heart Study (FHS)[13], cases where a FRS value was less than 1, values were rounded up by the package. Chronic kidney disease (CKD) included diagnoses of CKD secondary to lupus nephritis verified by Rheumatology clinicians. For patients with clinical trait data missing, a score was not obtainable (indicated as not applicable/NA).

Machine learning metabolomics analysis

Data cleaning and imputation: K-nearest neighbours [14] was used for imputation where missing data was <10%, this method uses the majority to determine classification of a new unknown case that has several features in common with existing cases using default parameters [15]. Metabolites with missing values >10% were excluded from the models due to avoid distorting the results. Metabolites were normalised using centre scaling prior to model fitting.

Classification and regression models: Logistic regression (LR) forest plot was applied to metabolomic and clinical data to predict plaque status in SLE patients from the Discovery cohort (SLE-P n=18, SLE-NP=26) based on the pipeline developed previously [6]. Homology reduction was applied to metabolite panels to reduce the risk of model overfitting (metabolites removed if >0.95 correlation). Five classification machine learning models (Least Absolute Shrinkage and Selection Operator (LASSO) LR, random forest (RF), neural network (NN), support vector machine (SVM) and eXtreme gradient boost (XGB) (caret package12 in R [https://cran.r-project.org/web/packages/caret/index.html]) were used to identify which metabolites and/or clinical features were important predictors of plaque status. Models were optimized to specific parameters [16] and 10-fold cross validation was conducted. Model performance was assessed using accuracy (area under the curve–receiver operator characteristic, ROC-AUC), sensitivity, specificity and an F1 score (derived from recall and precision). Classification models that performed with an accuracy (area under curve-receiver operating characteristic, AUC-ROC) >0.7 were included in developing a CVD-risk signature which retained any important feature creating a combined model signature (UCLH40).

Sparse partial least squares discriminant analysis (sPLS-da) was used to evaluate the derived signature with principal component analysis (PCA) indicating the separation of groups using features in the signature. The UCLH-40 CVD-risk signature was applied to the Validation cohort (SLE-P n=21, SLE-NP n=77, 100% female) and a JSLE cohort (JSLE-high n=19, 56%, JSLE-low CIMT progression n=15, 44%). Venn analysis was used to determine which features were common between published sets [4,6] and the UCLH40 signature.

Model metrics: Models were applied to classify SLE-P (n=18, true positives) and SLE-NP (n=26, true negatives) patient and were tuned using step-wise sequences to assess the optimal parameter for each model operation; 10-fold cross validation was implemented. For UCLH Discovery cohort: LR $\alpha=1$, $\lambda = 0.045$, RF mtry = 6, NN size = 4, decay = 0.5, SVM $\sigma=0.01$, C=5, XGB $\gamma = 0.1$. For Validation cohort: LR $\alpha=1$, $\lambda = 0.065$, RF mtry = 10, NN size = 2, decay = 0.1, SVM $\sigma=0.01$, C=5, XGB $\gamma = 0.1$. For Test cohort: LR $\alpha=1$, $\lambda = 0.038$, RF mtry = 3, NN size = 1, decay = 0.01, SVM $\sigma=0.01$, C=5, XGB $\gamma = 0.1$.

Atherosclerosis-risk score

A cut off score associated with plaque status was calculated for features in the UCLH signature using MetaboAnalyst biomarker detection algorithm [https://www.metaboanalyst.ca/] and Youden scores [17] that calculates an accuracy (ROC-AUC), sensitivity and specificity of the cut off value to correctly predict plaque status. Features in the signature were ranked by importance (ROC-AUC) and the number of features for required to assess plaque status was measured using AutoScore [18] in R [https://nliulab.github.io/AutoScore/04-autoscore.html] using a 30% training and 70% test dataset on the UCLH-40 signature using random forest. A training and testing model were both assessed for accuracy (ROC-AUC). The testing model was adapted to include only metabolites that had a high discriminatory accuracy (>=0.9 ROC-AUC). Model parameters were optimized using the Youden value. A points system was developed to score new patients based on the combined metabolite panel cut off scores where scores relate to likelihood of having subclinical plaque (% risk). Points are weighted to contributions of individual features.

Analysis of UK Biobank metabolomics

Values of the four metabolites making up the 5-panel score were analysed using the open access Nightingale Biomarker Disease Atlas tool [https://biomarker-atlas.nightingale.cloud/] which makes metabolomic and basic clinical outcome data from the UK BioBank available. Incidence of ‘acute myocardial infarction’ (Endpoint ICD-10 code I21), ‘stroke, not specified as haemorrhage or infarction’ (Endpoint ICD-10 code I64) and ‘other peripheral vascular diseases’ (Endpoint ICD-10 code I73) adjusted for markers within the 5-panel score -omega-6/omega-3, glycine, M-LDL-C and IDL-C concentrations were tested in the full population (all ages). Hazard ratios and p-values for each feature are reported.

Statistical analyses

Assessment of normality (Shapiro-Wilk normality test), correlation analyses (Pearson’s r or Spearman’s rho) and between group comparisons (unpaired t-test or Mann-Whitney U) were performed using R dplyr package [https://dplyr.tidyverse.org/index.html] Shapiro function and oneTable package [https://cran.r-project.org/web/packages/Tableone/index.html]. Clinical differences between cohorts were analysed using oneTable for statistical significance between SLE-P and SLE-NP [https://github.com/kaz-yos/tableone].

Data visualisation

Violin plots were created using R-4.2.1 for Windows [https://cran.r-project.org/bin/windows/base/] and ggplot2 [19] [https://ggplot2.tidyverse.org]. Figures were made using Adobe Illustrator [https://adobe.com/products/illustrator]. Logistic regression forest plots were visualised using Nightingale forest plot with ggplot2 [https://github.com/NightingaleHealth/ggforestplot.git].

Table S1. Atherosclerotic plaque characteristics-UCLH Discovery Cohort: Values taken from vascular ultrasound scans. Described in [1]. AUS, Aerial ultrasound score; L, left; R, right; IMT, intima-media thickness; GSM, Grey scale median.

	SLE_P	SLE_P	SLE_P	SLE_P	SLE_P	SLE_P	SLE_P	SLE_P	SLE_P	SLE_P	SLE_P	SLE_P	SLE_P	SLE_P	SLE_P	SLE_P	SLE_P	SLE_P
Plaque No.	2	2	2	2	2	2	1	2	1	1	2	4	2	3	4	1	4	1
R carotid AUS	4	6	8	6	2	6	2	2	6	0	6	6	6	2	6	6	6	2
L carotid AUS	4	2	8	6	6	6	6	2	2	0	6	6	6	6	6	0	6	2
R femoral AUS	4	2	2	2	2	2	2	6	2	6	2	6	2	6	6	2	6	6
L femoral AUS	4	6	2	2	6	2	2	6	4	4	4	6	2	6	6	2	6	2
Total AUS score	20	16	20	16	16	16	12	16	14	10	18	24	16	20	24	10	24	12
R carotid	0.09	0.07	0.08	0.06	0.05	0.06	0.05	0.05	0.06	0.07	0.07	0.06	0.06	0.08	0.08	0.05	0.07	0.07
R origin	0.14	0.23	0.42	0.19	0.11	0.26	0.11	0.12	0.02	0.11	0.2	0.28		0.1	0.32	0.35	0.39	0.1
L carotid IMT	0.08	0.06	0.09	0.08	0.04	0.06	0.06	0.06	0.06	0.07	0.08	0.06	0.06	0.07	0.07	0.06	0.06	0.06
L original	0.12	0.11	0.26	0.22	0.19	0.25	0.16	0.1	0.1	0.1	0.16	0.27		0.3	0.22	1	0.29	0.1
Mean carotid IMT	0.09	0.06	0.09	0.07	0.04	0.06	0.06	0.06	0.06	0.07	0.08	0.06	0.06	0.08	0.08	0.06	0.07	0.07
R carotid GSM			58	30		74			40		40	53	60		31	30	75	
L carotid GSM			109	39	73	39	36				136	105	30	53	21		72	
R femoral GSM	111							68		69		11		71	129		68	79
L femoral GSM	76				63			119				13		75	85		33	
Mean carotid GSM			83.5	34.5	73	56.5	36		40		88	79	45	53	26	30	73.5	
Mean femoral GSM	93.5				63			93.5		69		12		73	107		50.5	79
Mean GSM	93.5		83	34.5	68	56.5		93.5	40	69	88	45.5	45	62	66.5	30	62	79
R bulb area		16.6	70.7	4.4		10.3			11.9		11.8	19.7	11.3		25.2	80.3	9.59	
L bulb area			39.7	13.2	7.56	11.5	9.87				7.89	13.8	10.2	7.7	15.4		16.2	
R femoral area	48.6							22.9		32.8		89		50.5	9.5		55.2	18
L femoral area	37.1		98.9		19			27.6				23.7		39.6	43.1		30.5	
Total plaque area	85.7	75.5	110	33.5	26.6	21.8	9.87	50.5	11.9	32.8	19.7	146	21.5	97.8	93.2	80.3	111	18
R carotid thickness		0.27	0.42	0.21		0.26			0.23		0.2	0.33	0.2		0.32	0.41	0.39	
L carotid thickness			0.36	1.84	0.19	0.25	0.15				0.16	0.21	0.14	0.23	0.24		0.29	
L carotid length			2.23	1.82	0.47	0.78	1.03				0.81	0.95	1.1	0.55	0.89		0.94	
R femoral thickness	0.29							0.22		0.18		0.35		0.33	0.16		0.3	0.14
R femoral length	1.84							1.59		2.14		3.09		2.18	0.92		2.34	2.05
L femoral length	1.69				0.63			0.64				2.56		1.43	3.86		1.67	
L femoral thickness	0.29	0.2			0.28			0.2				0.25		0.27	0.2		0.29	
L femoral length	2.13	1.61			0.95			1.71				1.87		1.65	2.27		1.82	

Total plaque thickness	0.53	0.47	0.78	0.38	0.47	0.47	0.15	0.42	0.23	0.18	0.37	1.14	0.34	0.82	0.91	0.41	1.27	0.14
Total plaque length	3.97	3.41	4.04	2.57	1.41	1.69	1.03	3.3	0.91	2.14		6.68	2.01	4.38	5.54	2.9	5.79	2.05

Table S2. Metabolite Abbreviations: Abbreviations for full metabolite panel (Nightingale).

ABBREVIATION	DESCRIPTION
Total-C	Total cholesterol
non-HDL-C	Total cholesterol minus HDL-C
Remnant-C	Remnant cholesterol (non-HDL
VLDL-C	VLDL cholesterol
Clinical LDL-C	Clinical LDL cholesterol
LDL-C	LDL cholesterol
HDL-C	HDL cholesterol
HDL2-C	HDL2 cholesterol
HDL3-C	HDL3 cholesterol
Total-TG	Total triglycerides
VLDL-TG	Triglycerides in VLDL
LDL-TG	Triglycerides in LDL
HDL-TG	Triglycerides in HDL
Total-PL	Total phospholipids in lipoprotein particles
VLDL-PL	Phospholipids in VLDL
LDL-PL	Phospholipids in LDL
HDL-PL	Phospholipids in HDL
Total-CE	Total esterified cholesterol
VLDL-CE	Cholesteryl esters in VLDL
LDL-CE	Cholesteryl esters in LDL
HDL-CE	Cholesteryl esters in HDL
Total-FC	Total free cholesterol
VLDL-FC	Free cholesterol in VLDL
LDL-FC	Free cholesterol in LDL
HDL-FC	Free cholesterol in HDL
Total-L	Total lipids in lipoprotein particles
VLDL-L	Total lipids in VLDL
LDL-L	Total lipids in LDL
HDL-L	Total lipids in HDL
Total-P	Total concentration of lipoprotein particles
VLDL-P	Concentration of VLDL particles
LDL-P	Concentration of LDL particles
HDL-P	Concentration of HDL particles
VLDL size	Average diameter for VLDL particles
LDL size	Average diameter for LDL particles
HDL size	Average diameter for HDL particles
Phosphoglyc	Phosphoglycerides
TG/PG	Ratio of triglycerides to phosphoglycerides
Cholines	Total cholines
Phosphatidylc	Phosphatidylcholines
Sphingomyelins	Sphingomyelins
ApoB	Apolipoprotein B
ApoA1	Apolipoprotein A1

ABBREVIATION	DESCRIPTION
ApoB/ApoA1	apolipoprotein B:apolipoprotein A1
Total-FA	Total fatty acids
Unsaturation	Degree of unsaturation
Omega-3	Omega-3 fatty acids
Omega-6	Omega-6 fatty acids
PUFA	Polyunsaturated fatty acids
MUFA	Monounsaturated fatty acids
SFA	Saturated fatty acids
LA	Linoleic acid
DHA	Docosahexaenoic acid
Omega-3 %	Ratio of omega-3 fatty acids to total fatty acids
Omega-6 %	Ratio of omega-6 fatty acids to total fatty acids
PUFA %	Ratio of polyunsaturated fatty acids to total fatty acids
MUFA %	Ratio of monounsaturated fatty acids to total fatty acids
SFA %	Ratio of saturated fatty acids to total fatty acids
LA %	Ratio of linoleic acid to total fatty acids
DHA %	Ratio of docosahexaenoic acid to total fatty acids
PUFA/MUFA	Ratio of polyunsaturated fatty acids to monounsaturated fatty acids
Omega-6/Omega-3	Ratio of omega-6 fatty acids to omega-3 fatty acids
Ala	Alanine
Gln	Glutamine
Gly	Glycine
His	Histidine
Total BCAA	Total concentration of branched-chain amino acids (leucine + isoleucine + valine)
Ile	Isoleucine
Leu	Leucine
Val	Valine
Phe	Phenylalanine
Tyr	Tyrosine
Glucose	Glucose
Lactate	Lactate
Pyruvate	Pyruvate
Citrate	Citrate
Glycerol	Glycerol
bOHbutyrate	3-Hydroxybutyrate
Acetate	Acetate
Acetoacetate	Acetoacetate
Acetone	Acetone
Creatinine	Creatinine
Albumin	Albumin

GlycA	Glycoprotein acetyls
XXL-VLDL-P	Concentration of chylomicrons and extremely large VLDL particles
XXL-VLDL-L	Total lipids in chylomicrons and extremely large VLDL
XXL-VLDL-PL	Phospholipids in chylomicrons and extremely large VLDL
XXL-VLDL-C	Cholesterol in chylomicrons and extremely large VLDL
XXL-VLDL-CE	Cholesteryl esters in chylomicrons and extremely large VLDL
XXL-VLDL-FC	Free cholesterol in chylomicrons and extremely large VLDL
XXL-VLDL-TG	Triglycerides in chylomicrons and extremely large VLDL
XL-VLDL-P	Concentration of very large VLDL particles
XL-VLDL-L	Total lipids in very large VLDL
XL-VLDL-PL	Phospholipids in very large VLDL
XL-VLDL-C	Cholesterol in very large VLDL
XL-VLDL-CE	Cholesteryl esters in very large VLDL
XL-VLDL-FC	Free cholesterol in very large VLDL
XL-VLDL-TG	Triglycerides in very large VLDL
L-VLDL-P	Concentration of large VLDL particles
L-VLDL-L	Total lipids in large VLDL
L-VLDL-PL	Phospholipids in large VLDL
L-VLDL-C	Cholesterol in large VLDL
L-VLDL-CE	Cholesteryl esters in large VLDL
L-VLDL-FC	Free cholesterol in large VLDL
L-VLDL-TG	Triglycerides in large VLDL
M-VLDL-P	Concentration of medium VLDL particles
M-VLDL-L	Total lipids in medium VLDL
M-VLDL-PL	Phospholipids in medium VLDL
M-VLDL-C	Cholesterol in medium VLDL
M-VLDL-CE	Cholesteryl esters in medium VLDL
M-VLDL-FC	Free cholesterol in medium VLDL
M-VLDL-TG	Triglycerides in medium VLDL
S-VLDL-P	Concentration of small VLDL particles
S-VLDL-L	Total lipids in small VLDL
S-VLDL-PL	Phospholipids in small VLDL
S-VLDL-C	Cholesterol in small VLDL
S-VLDL-CE	Cholesteryl esters in small VLDL
S-VLDL-FC	Free cholesterol in small VLDL
S-VLDL-TG	Triglycerides in small VLDL
XS-VLDL-P	Concentration of very small VLDL particles
XS-VLDL-L	Total lipids in very small VLDL
XS-VLDL-PL	Phospholipids in very small VLDL
XS-VLDL-C	Cholesterol in very small VLDL
XS-VLDL-CE	Cholesteryl esters in very small VLDL
XS-VLDL-FC	Free cholesterol in very small VLDL

XS-VLDL-TG	Triglycerides in very small VLDL
IDL-P	Concentration of IDL particles
IDL-L	Total lipids in IDL
IDL-PL	Phospholipids in IDL
IDL-C	Cholesterol in IDL
IDL-CE	Cholesteryl esters in IDL
IDL-FC	Free cholesterol in IDL
IDL-TG	Triglycerides in IDL
L-LDL-P	Concentration of large LDL particles
L-LDL-L	Total lipids in large LDL
L-LDL-PL	Phospholipids in large LDL
L-LDL-C	Cholesterol in large LDL
L-LDL-CE	Cholesteryl esters in large LDL
L-LDL-FC	Free cholesterol in large LDL
L-LDL-TG	Triglycerides in large LDL
M-LDL-P	Concentration of medium LDL particles
M-LDL-L	Total lipids in medium LDL
M-LDL-PL	Phospholipids in medium LDL
M-LDL-C	Cholesterol in medium LDL
M-LDL-CE	Cholesteryl esters in medium LDL
M-LDL-FC	Free cholesterol in medium LDL
M-LDL-TG	Triglycerides in medium LDL
S-LDL-P	Concentration of small LDL particles
S-LDL-L	Total lipids in small LDL
S-LDL-PL	Phospholipids in small LDL
S-LDL-C	Cholesterol in small LDL
S-LDL-CE	Cholesteryl esters in small LDL
S-LDL-FC	Free cholesterol in small LDL
S-LDL-TG	Triglycerides in small LDL
XL-HDL-P	Concentration of very large HDL particles
XL-HDL-L	Total lipids in very large HDL
XL-HDL-PL	Phospholipids in very large HDL
XL-HDL-C	Cholesterol in very large HDL
XL-HDL-CE	Cholesteryl esters in very large HDL
XL-HDL-FC	Free cholesterol in very large HDL
XL-HDL-TG	Triglycerides in very large HDL
L-HDL-P	Concentration of large HDL particles
L-HDL-L	Total lipids in large HDL
L-HDL-PL	Phospholipids in large HDL
L-HDL-C	Cholesterol in large HDL
L-HDL-CE	Cholesteryl esters in large HDL
L-HDL-FC	Free cholesterol in large HDL
L-HDL-TG	Triglycerides in large HDL
M-HDL-P	Concentration of medium HDL particles
M-HDL-L	Total lipids in medium HDL
M-HDL-PL	Phospholipids in medium HDL

M-HDL-C	Cholesterol in medium HDL
M-HDL-CE	Cholesteryl esters in medium HDL
M-HDL-FC	Free cholesterol in medium HDL
M-HDL-TG	Triglycerides in medium HDL
S-HDL-P	Concentration of small HDL particles
S-HDL-L	Total lipids in small HDL
S-HDL-PL	Phospholipids in small HDL
S-HDL-C	Cholesterol in small HDL
S-HDL-CE	Cholesteryl esters in small HDL
S-HDL-FC	Free cholesterol in small HDL
S-HDL-TG	Triglycerides in small HDL
XXL-VLDL-PL %	Phospholipids to total lipids ratio in chylomicrons and extremely large VLDL
XXL-VLDL-C %	Cholesterol to total lipids ratio in chylomicrons and extremely large VLDL
XXL-VLDL-CE %	Cholesteryl esters to total lipids ratio in chylomicrons and extremely large VLDL
XXL-VLDL-FC %	Free cholesterol to total lipids ratio in chylomicrons and extremely large VLDL
XXL-VLDL-TG %	Triglycerides to total lipids ratio in chylomicrons and extremely large VLDL
XL-VLDL-PL %	Phospholipids to total lipids ratio in very large VLDL
XL-VLDL-C %	Cholesterol to total lipids ratio in very large VLDL
XL-VLDL-CE %	Cholesteryl esters to total lipids ratio in very large VLDL
XL-VLDL-FC %	Free cholesterol to total lipids ratio in very large VLDL
XL-VLDL-TG %	Triglycerides to total lipids ratio in very large VLDL
L-VLDL-PL %	Phospholipids to total lipids ratio in large VLDL
L-VLDL-C %	Cholesterol to total lipids ratio in large VLDL
L-VLDL-CE %	Cholesteryl esters to total lipids ratio in large VLDL
L-VLDL-FC %	Free cholesterol to total lipids ratio in large VLDL
L-VLDL-TG %	Triglycerides to total lipids ratio in large VLDL
M-VLDL-PL %	Phospholipids to total lipids ratio in medium VLDL
M-VLDL-C %	Cholesterol to total lipids ratio in medium VLDL
M-VLDL-CE %	Cholesteryl esters to total lipids ratio in medium VLDL
M-VLDL-FC %	Free cholesterol to total lipids ratio in medium VLDL
M-VLDL-TG %	Triglycerides to total lipids ratio in medium VLDL
S-VLDL-PL %	Phospholipids to total lipids ratio in small VLDL
S-VLDL-C %	Cholesterol to total lipids ratio in small VLDL

S-VLDL-CE %	Cholesteryl esters to total lipids ratio in small VLDL
S-VLDL-FC %	Free cholesterol to total lipids ratio in small VLDL
S-VLDL-TG %	Triglycerides to total lipids ratio in small VLDL
XS-VLDL-PL %	Phospholipids to total lipids ratio in very small VLDL
XS-VLDL-C %	Cholesterol to total lipids ratio in very small VLDL
XS-VLDL-CE %	Cholesteryl esters to total lipids ratio in very small VLDL
XS-VLDL-FC %	Free cholesterol to total lipids ratio in very small VLDL
XS-VLDL-TG %	Triglycerides to total lipids ratio in very small VLDL
IDL-PL %	Phospholipids to total lipids ratio in IDL
IDL-C %	Cholesterol to total lipids ratio in IDL
IDL-CE %	Cholesteryl esters to total lipids ratio in IDL
IDL-FC %	Free cholesterol to total lipids ratio in IDL
IDL-TG %	Triglycerides to total lipids ratio in IDL
L-LDL-PL %	Phospholipids to total lipids ratio in large LDL
L-LDL-C %	Cholesterol to total lipids ratio in large LDL
L-LDL-CE %	Cholesteryl esters to total lipids ratio in large LDL
L-LDL-FC %	Free cholesterol to total lipids ratio in large LDL
L-LDL-TG %	Triglycerides to total lipids ratio in large LDL
M-LDL-PL %	Phospholipids to total lipids ratio in medium LDL
M-LDL-C %	Cholesterol to total lipids ratio in medium LDL
M-LDL-CE %	Cholesteryl esters to total lipids ratio in medium LDL
M-LDL-FC %	Free cholesterol to total lipids ratio in medium LDL
M-LDL-TG %	Triglycerides to total lipids ratio in medium LDL
S-LDL-PL %	Phospholipids to total lipids ratio in small LDL
S-LDL-C %	Cholesterol to total lipids ratio in small LDL
S-LDL-CE %	Cholesteryl esters to total lipids ratio in small LDL
S-LDL-FC %	Free cholesterol to total lipids ratio in small LDL
S-LDL-TG %	Triglycerides to total lipids ratio in small LDL
XL-HDL-PL %	Phospholipids to total lipids ratio in very large HDL
XL-HDL-C %	Cholesterol to total lipids ratio in very large HDL
XL-HDL-CE %	Cholesteryl esters to total lipids ratio in very large HDL
XL-HDL-FC %	Free cholesterol to total lipids ratio in very large HDL

XL-HDL-TG %	Triglycerides to total lipids ratio in very large HDL		M-HDL-CE %	Cholesteryl esters to total lipids ratio in medium HDL
L-HDL-PL %	Phospholipids to total lipids ratio in large HDL		M-HDL-FC %	Free cholesterol to total lipids ratio in medium HDL
L-HDL-C %	Cholesterol to total lipids ratio in large HDL		M-HDL-TG %	Triglycerides to total lipids ratio in medium HDL
L-HDL-CE %	Cholesteryl esters to total lipids ratio in large HDL		S-HDL-PL %	Phospholipids to total lipids ratio in small HDL
L-HDL-FC %	Free cholesterol to total lipids ratio in large HDL		S-HDL-C %	Cholesterol to total lipids ratio in small HDL
L-HDL-TG %	Triglycerides to total lipids ratio in large HDL		S-HDL-CE %	Cholesteryl esters to total lipids ratio in small HDL
M-HDL-PL %	Phospholipids to total lipids ratio in medium HDL		S-HDL-FC %	Free cholesterol to total lipids ratio in small HDL
M-HDL-C %	Cholesterol to total lipids ratio in medium HDL		S-HDL-TG %	Triglycerides to total lipids ratio in small HDL

Table S3. Performance metrics for ML serum metabolomics analysis

Performance metrics for ML models applied to serum metabolomics using homology reduction. AUC-ROC, area under the curve-receiver operating characteristics; F1, Composite score of several metrics; LR, logistic regression; RF, random forest; NN, neural network; SVM, support vector machine; XGB, extreme gradient boost.

UCLH	ROC-AUC	Sensitivity	Specificity	Precision	Recall	F1
**LR**	0.90	0.88	0.78	0.52	0.82	0.64
RF	0.76	0.62	0.72	0.62	0.57	0.59
NN	0.72	0.73	0.67	0.48	0.63	0.55
SVM	0.59	0.58	0.61	0.50	0.50	0.50
XGB	**0.91**	0.81	0.89	0.70	0.76	0.73

Table S4. Athens validation cohort – SLE patient clinical and demographic characteristics

Table of demographics and disease characteristics for independent Validation cohort between SLE-P and SLE-NP patients. Statistical significance assessed by T tests and #Wilcoxon rank sum for non-parametric testing.

	SLE-NP	SLE-P	p
**n**	77	21	
**Sex (% Female)**	100	100	
**Age (mean (SD))**	43.04 (10.66)	52.90 (8.27)	<0.001
**Disease duration (median [IQR])**	5.00 [3.00, 12.00]	11.00 [8.00, 15.00]	0.04#
**SLEDAI (median [IQR])**	2.00 [0.00, 4.00]	2.00 [2.00, 4.00]	0.47#
**MAP (NR 70-100mm/Hg) (median [IQR])**	75.00 [69.00, 81.00]	79.00 [71.00, 84.00]	0.17#
**HCQ (n)**	67	18	0.88#
**Statins (n)**	8	4	0.29#
**ACE Inhibitors (n)**	7	5	0.07#
**Immunosuppressives (n)**	27	9	0.51#
**Rituximab (n)**	1	0	0.6#
**Aspirin (n)**	20	3	0.27#
**Prednisone (n)**	39	14	0.22#
**Prednisone dosage mg/d (median [IQR])**	0.00 [0.00, 7.50]	5.00 [0.00, 5.00]	0.49#
**No treatment (n)**	0	4	0.29#

Table S5. Validation of UCLH metabolite signature in Athens cohort

Performance metrics for UCLH-40 signature in the Athens-validation cohort. Age at diagnosis values were not available so not included in this analysis.

	ROC	Sensitivity	Specificity	Precision	Recall	F1
**LR**	0.78	0.96	0.29	0.07	0.67	0.12
**RF**	0.68	0.60	0.71	0.29	0.33	0.31
**NN**	0.72	0.69	0.67	0.23	0.37	0.29
**SVM**	0.70	0.58	0.67	0.27	0.30	0.29
**XGB**	0.79	0.74	0.62	0.20	0.39	0.27

Table S6. Calculation of 5-panel cut points using Youden Index. The cut-point scores (distinguishing between SLE-P/SLE-NP) for each feature selected by the parsimony plot (Figure 3D) were calculated using the Youden Index. Cut off scores and performance metrics are shown for top features in 5-panel signature, cut-off represents metabolite concentration. AUC-ROC, Area under the curve-Receiver operating characteristics; M-LDL-C, medium-low density lipoprotein- cholesterol; IDL-C, intermediate density lipoprotein-cholesterol.

	Age	Glycine	M-LDL-C	IDL-C	Omega-6/Omega-3
**AUC-ROC**	0.853	0.812	0.78	0.774	0.641
**Cut off**	51.5	0.353	0.487	1.02	9.38
**Sensitivity**	0.889 (0.722-1)	0.944 (0.778-1)	0.778 (0.556-0.944)	0.611 (0.386-0.833)	0.611 (0.444-0.833)
**Specificity**	0.769 (0.615-0.905)	0.615 (0.403-0.789)	0.692 (0.538-0.866)	0.769 (0.634-0.923)	0.692 (0.538-0.885)

Table S7. Metabolite panel points system. Points system for 5-panel score weighted according to feature importance. Calculated using Autoscore algorithm (Supplementary Methods). An optimal total threshold score of ≥7 was associated with a high risk for subclinical plaque was calculated on the training dataset (30% split UCLH Discovery cohort) and could be used to stratify plaque status with a combined accuracy of 80% (AUC-ROC 0.80, 95% CI 0.5278 – 1) using an RF model on the remaining test data.

Feature	Cut point	Score
**Glycine**	<0.293	4
	[0.293,0.338)	2
	[0.338,0.396)	2
	>=0.396	0
**Age**	<51.5	0
	[51.5,57)	0
	>=57	2
**M-LDL-C**	<0.592	0
	>=0.592	1
**IDL-C**	<0.811	0
	[0.811,1.02)	1
	[1.02,1.17)	1
	>=1.17	2
**Omega-6/Omega-3**	<7.88	2
	[7.88,10.1)	2
	[10.1,15.4)	0
	>=15.4	0

Table S8. 5-panel risk scores in adult SLE: Discovery UCLH and Validation Athens cohort risk scores using 5-feature panel.

Cohort	Phenotype	Risk score	Risk group
Athens	SLE-NP	0	Low
Athens	SLE-NP	8	High
Athens	SLE-NP	3	Low
Athens	SLE-P	5	Low
Athens	SLE-NP	2	Low
Athens	SLE-NP	3	Low
Athens	SLE-NP	1	Low
Athens	SLE-NP	2	Low
Athens	SLE-NP	8	High
Athens	SLE-P	5	Low
Athens	SLE-NP	3	Low
Athens	SLE-NP	7	High
Athens	SLE-NP	9	High
Athens	SLE-NP	6	Low
Athens	SLE-NP	2	Low
Athens	SLE-NP	5	Low
Athens	SLE-NP	5	Low
Athens	SLE-NP	5	Low
Athens	SLE-NP	3	Low
Athens	SLE-NP	4	Low
Athens	SLE-NP	6	Low
Athens	SLE-NP	6	Low
Athens	SLE-NP	0	Low
Athens	SLE-NP	3	Low
Athens	SLE-NP	4	Low
Athens	SLE-P	6	Low
Athens	SLE-NP	1	Low
Athens	SLE-P	5	Low
Athens	SLE-NP	4	Low
Athens	SLE-NP	5	Low
Athens	SLE-P	5	Low
Athens	SLE-NP	1	Low
Athens	SLE-P	1	Low
Athens	SLE-NP	5	Low
Athens	SLE-NP	2	Low
Athens	SLE-NP	6	Low
Athens	SLE-P	6	Low
Athens	SLE-NP	6	Low
Athens	SLE-NP	3	Low
Athens	SLE-NP	5	Low
Athens	SLE-NP	3	Low
Athens	SLE-P	7	Low
Athens	SLE-NP	6	Low
Athens	SLE-NP	2	Low
Athens	SLE-P	9	High
Athens	SLE-NP	8	High
Athens	SLE-NP	5	Low
Athens	SLE-NP	3	Low
Athens	SLE-NP	8	High
Athens	SLE-NP	5	Low
Athens	SLE-NP	2	Low
Athens	SLE-NP	5	Low
Athens	SLE-NP	5	Low
Athens	SLE-P	4	Low
Athens	SLE-NP	4	Low
Athens	SLE-NP	3	Low
Athens	SLE-P	3	Low
Athens	SLE-P	3	Low
Athens	SLE-NP	3	Low
Athens	SLE-NP	4	Low
Athens	SLE-NP	9	High
Athens	SLE-NP	3	Low
Athens	SLE-P	8	High
Athens	SLE-P	2	Low
Athens	SLE-NP	2	Low
Athens	SLE-NP	5	Low
Athens	SLE-NP	5	Low
Athens	SLE-NP	5	Low
Athens	SLE-NP	3	Low
Athens	SLE-NP	7	High
Athens	SLE-NP	3	Low
Athens	SLE-NP	5	Low
Athens	SLE-NP	3	Low
Athens	SLE-NP	5	Low
Athens	SLE-NP	5	Low
Athens	SLE-NP	4	Low
Athens	SLE-P	4	Low
Athens	SLE-P	3	Low
Athens	SLE-NP	3	Low
Athens	SLE-P	5	Low
UCLH	SLE-P	10	High
UCLH	SLE-P	7	High
UCLH	SLE-NP	6	Low
UCLH	SLE-P	7	High
UCLH	SLE-P	9	High
UCLH	SLE-NP	5	Low
UCLH	SLE-P	3	Low
UCLH	SLE-NP	5	Low
UCLH	SLE-NP	3	Low
UCLH	SLE-P	7	High
UCLH	SLE-P	7	High
UCLH	SLE-P	5	Low
UCLH	SLE-NP	2	Low
UCLH	SLE-NP	6	Low
UCLH	SLE-P	6	Low
UCLH	SLE-NP	5	Low
UCLH	SLE-P	7	High
UCLH	SLE-NP	2	Low
UCLH	SLE-P	9	High
UCLH	SLE-NP	3	Low
UCLH	SLE-NP	4	Low
UCLH	SLE-NP	0	Low
UCLH	SLE-NP	2	Low
UCLH	SLE-NP	2	Low
UCLH	SLE-NP	5	Low
UCLH	SLE-P	7	High

Athens	SLE-NP	4	Low
Athens	SLE-NP	3	Low
Athens	SLE-P	2	Low
Athens	SLE-NP	4	Low
Athens	SLE-P	5	Low
Athens	SLE-P	7	High
Athens	SLE-NP	3	Low
Athens	SLE-NP	5	Low
Athens	SLE-NP	7	Low
Athens	SLE-NP	5	Low
Athens	SLE-NP	6	Low
Athens	SLE-NP	2	Low
Athens	SLE-P	9	High
Athens	SLE-NP	2	Low
Athens	SLE-NP	7	High
Athens	SLE-NP	6	Low
Athens	SLE-NP	7	High
Athens	SLE-NP	2	Low

UCLH	SLE-NP	5	Low
UCLH	SLE-P	7	High
UCLH	SLE-NP	6	Low
UCLH	SLE-NP	3	Low
UCLH	SLE-P	7	High
UCLH	SLE-NP	2	Low
UCLH	SLE-P	7	High
UCLH	SLE-NP	3	Low
UCLH	SLE-NP	4	Low
UCLH	SLE-NP	5	Low
UCLH	SLE-P	5	Low
UCLH	SLE-NP	5	Low
UCLH	SLE-P	3	Low
UCLH	SLE-NP	2	Low
UCLH	SLE-P	8	High
UCLH	SLE-NP	1	Low
UCLH	SLE-NP	1	Low
UCLH	SLE-NP	1	Low

Figure S1

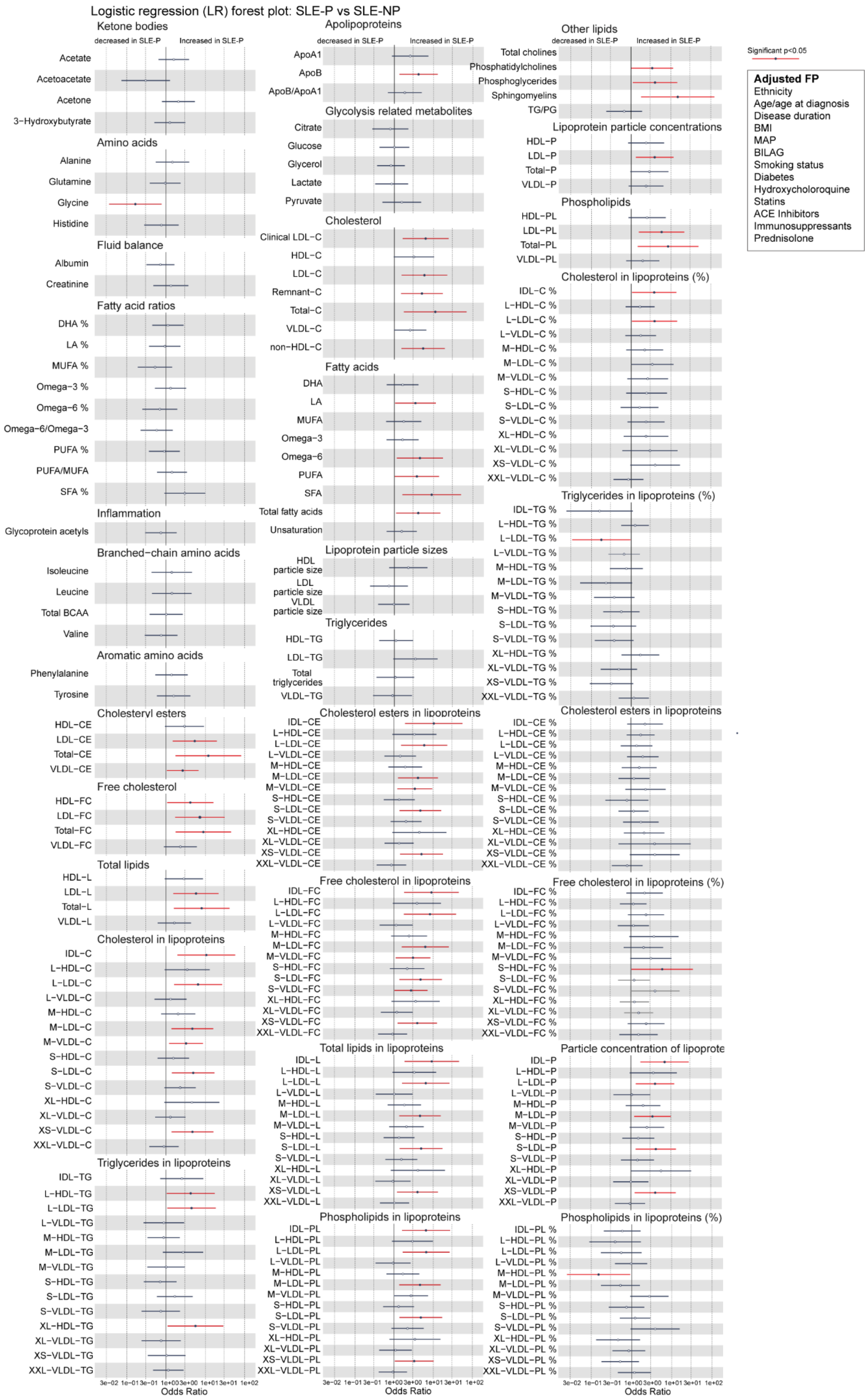
Logistic regression (LR) forest plot: SLE-P vs SLE-NP
Ketone bodies
Amino acids
Fluid balance
Fatty acid ratios
Inflammation
Branched−chain amino acids
Aromatic amino acids
Cholesteryl esters
Free cholesterol
Total lipids
Cholesterol in lipoproteins
Triglycerides in lipoproteins
Apolipoproteins
Glycolysis related metabolites
Cholesterol
Fatty acids
Lipoprotein particle sizes
Triglycerides
Cholesterol esters in lipoproteins
Free cholesterol in lipoproteins
Total lipids in lipoproteins
Phospholipids in lipoproteins
Other lipids
Lipoprotein particle concentrations
Phospholipids
Cholesterol in lipoproteins (%)
Triglycerides in lipoproteins (%)
Cholesterol esters in lipoproteins
Free cholesterol in lipoproteins (%)
Particle concentration of lipoprote
Phospholipids in lipoproteins (%)
decreased in SLE-P
Increased in SLE-P
Odds Ratio
Significant p<0.05
Adjusted FP
Ethnicity
Age/age at diagnosis
Disease duration
BMI
MAP
BILAG
Smoking status
Diabetes
Hydroxycholoroquine
Statins
ACE Inhibitors
Immunosuppressants
Prednisolone

Figure S1: Forest plot showing metabolomics univariate LR analysis of UCLH Discovery Cohort. Adjusted for several clinical traits, including disease activity and treatment (indicated) using LR model in SLE-P vs SLE-NP for 250 metabolite panel. Filled circle indicates statistical significance ($p<0.05$), unfilled indicates no difference.

Figure S2

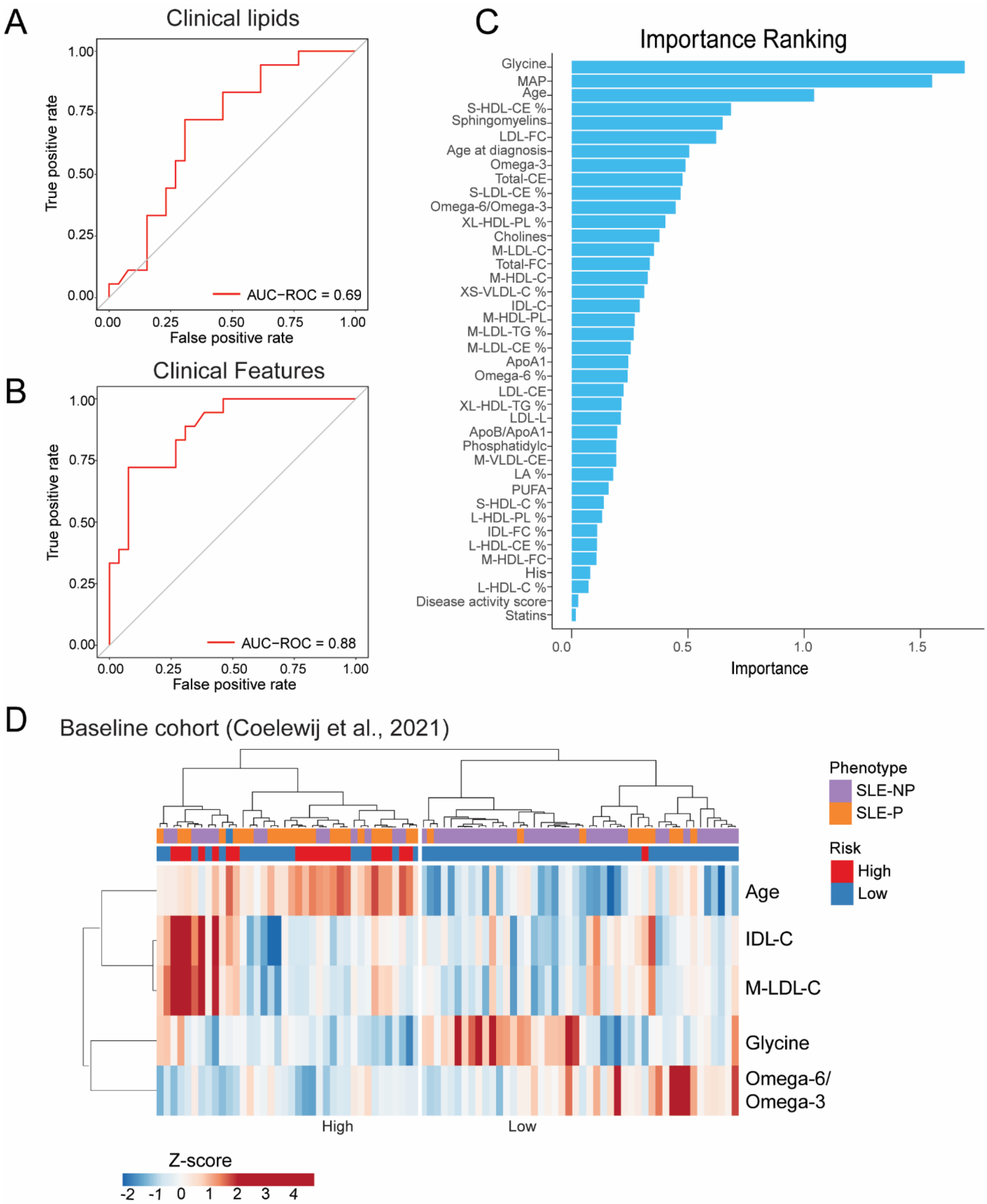

Figure S2. Validation of atherosclerosis-risk scores. ROC indicating accuracy of **A**. clinical laboratory lipid assessment and **B.** clinical features age, MAP, diabetes, smoking, and disease activity, applied to UCLH discovery using XGB model**. C.** Importance plot ranking metabolites and clinical features by importance (features from UCLH40 signature with importance ranking >0 plotted)**. D.** Hierarchical clustering heatmap

of 5-feature CVD risk score applied to metabolite concentrations of features obtained in a separate analysis ~5 years prior [6]). Plaque and risk groups indicated.

Figure S3

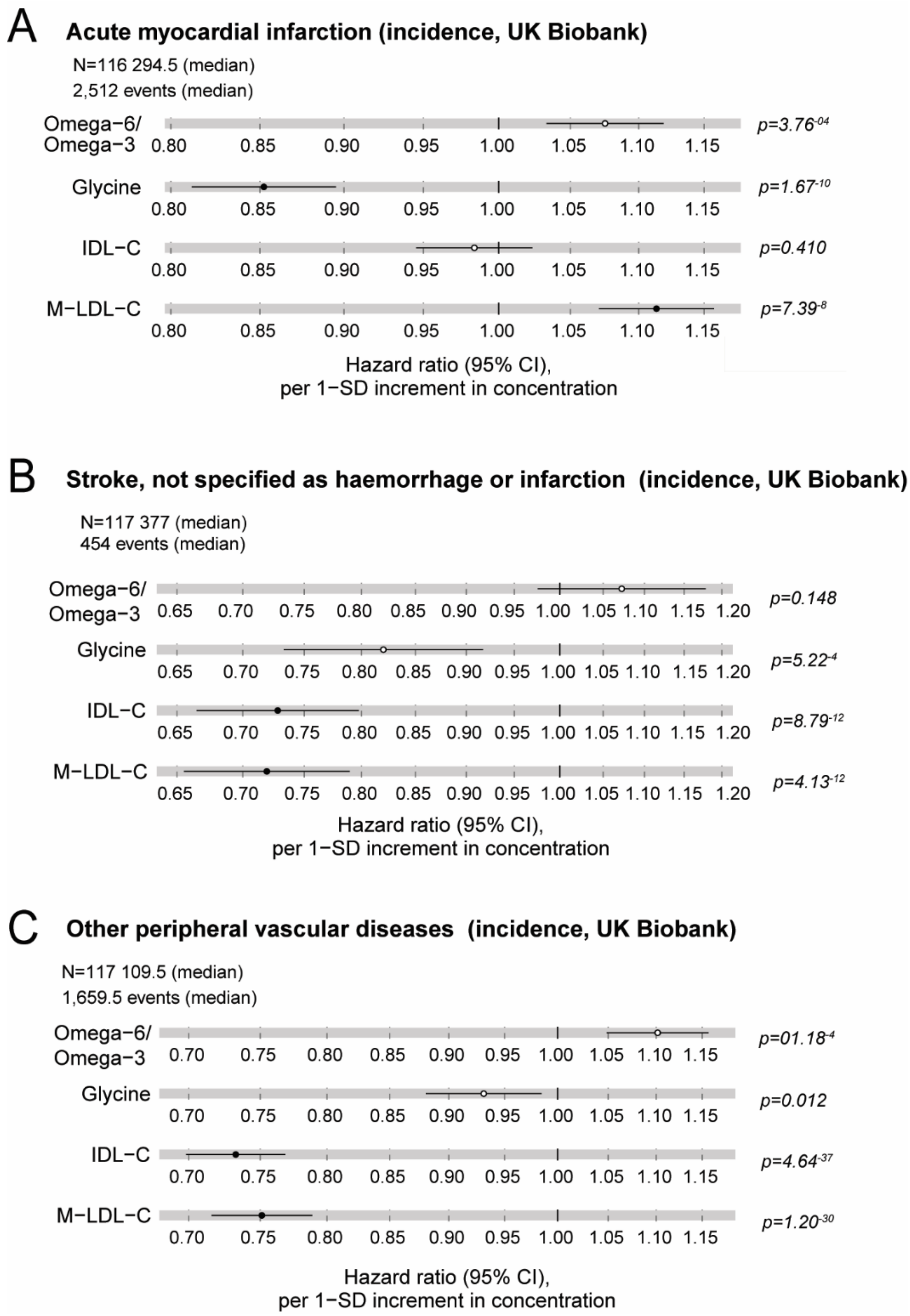

Figure S3: Incidence of **(A)** acute myocardial infarction (Endpoint ICD-10 code I21), **(B)** Stroke, not specified as haemorrhage or infarction (Endpoint ICD-10 code I64) and **(C)** Other peripheral vascular diseases (Endpoint ICD-10 code I73) adjusted for omega-6/omega-3, glycine, M-LDL-C and IDL-C concentrations in the full UK Biobank population were tested (all ages). Hazard ratios and p-values for each feature are reported.

1. Croca SC, Griffin M, Farinha F, Isenberg DA, Nicolaides A, Rahman A. Total plaque area and plaque echogenicity are novel measures of subclinical atherosclerosis in patients with systemic lupus erythematosus. *Rheumatology (Oxford)*. Sep 1 2021;60(9):4185-4198. doi:10.1093/rheumatology/keaa905
2. Schoenfeld SR, Kasturi S, Costenbader KH. The epidemiology of atherosclerotic cardiovascular disease among patients with SLE: a systematic review. *Semin Arthritis Rheum*. Aug 2013;43(1):77-95. doi:10.1016/j.semarthrit.2012.12.002
3. Tektonidou MG, Kravvariti E, Konstantonis G, Tentolouris N, Sfikakis PP, Protogerou A. Subclinical atherosclerosis in Systemic Lupus Erythematosus: Comparable risk with Diabetes Mellitus and Rheumatoid Arthritis. *Autoimmun Rev*. Mar 2017;16(3):308-312. doi:10.1016/j.autrev.2017.01.009
4. Peng J, Dönnes P, Ardoin SP, et al. Atherosclerosis Progression in the APPLE Trial Can Be Predicted in Young People With Juvenile-Onset Systemic Lupus Erythematosus Using a Novel Lipid Metabolomic Signature. *Arthritis Rheumatol*. Oct 2 2023;doi:10.1002/art.42722
5. Schanberg LE, Sandborg C, Barnhart HX, et al. Premature atherosclerosis in pediatric systemic lupus erythematosus: risk factors for increased carotid intima-media thickness in the atherosclerosis prevention in pediatric lupus erythematosus cohort. *Arthritis Rheum*. May 2009;60(5):1496-507. doi:10.1002/art.24469
6. Coelewij L, Waddington KE, Robinson GA, et al. Serum Metabolomic Signatures Can Predict Subclinical Atherosclerosis in Patients With Systemic Lupus Erythematosus. *Arterioscler Thromb Vasc Biol*. Apr 2021;41(4):1446-1458. doi:10.1161/atvbaha.120.315321
7. Isenberg DA, Rahman A, Allen E, et al. BILAG 2004. Development and initial validation of an updated version of the British Isles Lupus Assessment Group's disease activity index for patients with systemic lupus erythematosus. *Rheumatology (Oxford)*. Jul 2005;44(7):902-6. doi:10.1093/rheumatology/keh624
8. Zhang X, Li Y, Wang Y, et al. Contribution of serum lipids as effect modifiers to a relationship between mean arterial pressure and coronary heart disease in Chinese rural population: the Henan Rural Cohort Study. *BMJ Open*. Nov 7 2019;9(11):e029179. doi:10.1136/bmjopen-2019-029179
9. Gladman DD. Indicators of disease activity, prognosis, and treatment of systemic lupus erythematosus. *Curr Opin Rheumatol*. Sep 1994;6(5):487-92. doi:10.1097/00002281-199409000-00006
10. Choi MY, Ma C. Making a big impact with small datasets using machine-learning approaches. *The Lancet Rheumatology*. 2020;2(8):e451-e452. doi:10.1016/S2665-9913(20)30217-4
11. Hippisley-Cox J, Coupland C, Brindle P. Development and validation of QRISK3 risk prediction algorithms to estimate future risk of cardiovascular disease: prospective cohort study. *Bmj*. May 23 2017;357:j2099. doi:10.1136/bmj.j2099
12. Li Y, Sperrin M and van Staa T. R package "QRISK3": an unofficial research purposed implementation of ClinRisk's QRISK3 algorithm into R [version 3; peer review: 1 approved, 1 approved with reservations, 1 not approved]. F1000Research 2020, 8:2139 (https://doi.org/10.12688/f1000research.21679.3)
13. D'Agostino RB, Sr., Vasan RS, Pencina MJ, et al. General cardiovascular risk profile for use in primary care: the Framingham Heart Study. *Circulation*. Feb 12 2008;117(6):743-53. doi:10.1161/circulationaha.107.699579
14. Hastie T, Tibshirani R, Sherlock G, Eisen M, Brown P, Botstein D. Imputing Missing Data for Gene Expression Arrays. 1999
15. Kuhn M. Classification and Regression Training [R package caret version 6.0-93]. 2022 Aug 9 [cited 2022 Dec 21]; Available from: https://CRAN.R-project.org/package=caret
16. Kuhn M. Building Predictive Models in R Using the caret Package. J Stat Softw [Internet]. 2008 Nov 10 [cited 2023 Jul 24];28:1–26. Available from: https://www.jstatsoft.org/index.php/jss/article/view/v028i05
17. Chong J, Xia J. Using MetaboAnalyst 4.0 for Metabolomics Data Analysis, Interpretation, and Integration with Other Omics Data. *Methods Mol Biol*. 2020;2104:337-360. doi:10.1007/978-1-0716-0239-3_17
18. Xie F, Chakraborty B, Ong MEH, Goldstein BA, Liu N. AutoScore: A Machine Learning-Based Automatic Clinical Score Generator and Its Application to Mortality Prediction Using Electronic Health Records. *JMIR Med Inform*. Oct 21 2020;8(10):e21798. doi:10.2196/21798
19. Wickham H. Ggplot2 : elegant graphics for data analysis [Internet]. Springer-Verlag New York; 2016 [cited 2022 Oct 19]. Available from: https://ggplot2.tidyverse.org/
